# Suppression of TGF-β1 signaling by Matrigel via FAK signaling in cultured human trabecular meshwork cells

**DOI:** 10.1038/s41598-021-86591-7

**Published:** 2021-04-01

**Authors:** Yuan Zhang, Scheffer C. G. Tseng, Ying-Ting Zhu

**Affiliations:** Research and Development Department, Tissue Tech, Inc., 7235 Corporate Center Drive, Suite B, Miami, FL 33172 USA

**Keywords:** Eye diseases, Cell signalling

## Abstract

The trabecular meshwork (TM) is composed of TM cells and beams of the extracellular matrix, together contributing to aqueous humor (AH) outflow resistance. Herein, we validated that our culture system on 2D Matrigel expressed putative TM markers and myocilin, of which the latter was upregulated by dexamethasone. Continuous passage of these cells on 2D Matrigel resulted in a gradual loss of expression of these markers. However, such a loss was restored by seeding cells in 3D Matrigel where expression of TM markers was further upregulated upon continuous passage. In contrast, TM cells seeded on fibronectin, collagen I/IV, or laminin lost expression of these markers and turned into myofibroblasts with expression of αSMA, which were dose-dependently upregulated by TGF-β1/TGF-β2. TM cells in 3D Matrigel also expressed TGF-β1/TGF-β3 despite challenge of TGF-β1. The maintenance of TM phenotype by 3D Matrigel was linked to inhibition of canonical TGF-β signaling and activation of pFAK-pSrc-pP190RhoGAP-P120RasGAP signaling. These findings indicate that basement membrane matrix with low rigidity plays an active role in maintaining TM phenotype in the presence of TGF-β1 and shed light on its physiological role. Furthermore, abnormal matrices may perpetuate the pathological TM phenotype when the level of TGF-β2 is elevated in glaucoma patients.

## Introduction

Glaucoma is an optic neuropathy defined by characteristic optic nerve damage and progressive visual field loss with elevated intraocular pressure (IOP) as a major risk factor (reviewed in^1–4^). The trabecular meshwork (TM) is composed of endothelial-like TM cells and beams of the extracellular matrix (ECM)^5^ and this conventional outflow pathway provides considerable resistance to aqueous outflow^6–8^. Therefore, the normal TM cell phenotype and the homeostatic control of synthesis and degradation of ECM play an important role in regulating IOP in normal eyes^9–12^. Altered TM phenotype with reduced cellularity/senescence and accumulation of altered ECM within TM contributes to increased resistance in TM and elevated IOP in glaucomatous eyes^13–16^.

Among growth factors/cytokines, transforming growth factor-beta (TGF-β) is the prime candidate contributing to the pathological state of increased outflow resistance in TM^17^. TGF-β2 is the major one among the three TGF-β isoforms^18,19^, present at the concentration of 0.41 to 2.24 ng/ml in normal human AH^20^, responsible for some physiological activities^20–22^. A significantly elevated level of TGF-β2 ranging from 1.94 to 3.46 ng/ml has been found in AH of patients with primary open angle glaucoma^20^. An elevated level of TGF-β2 is believed to contribute to elevated IOP and subsequent glaucoma by promoting excessive synthesis and deposition of matrix components such as collagen IV, fibronectin, laminin and elastin in TM^23–26^. It remains unclear whether the level of TGF-β in AH dictates its physiological or pathological role or there is an unknown mechanism that maintains a normal TM phenotype by withstanding the potential pathogenic impact from an elevated level of TGF-β in AH.

As a first step to resolve the aforementioned seemingly paradoxical (physiological vs. pathological) role of TGF-β in TM cells, we have successfully established a model system to isolate and expand human TM progenitor cells on two–dimensional (2D) Matrigel in modified embryonic stem cell medium (MESCM) containing 5% fetal bovine serum (FBS)^27^. As reviewed by Stamer et al^28^ and Keller et al^29^, the detection of a single TM marker is elusive and scientists rely on a panel of markers to identify TM cells, including MGP^30–32^, CHI3L1^32^, AQP1^32,33^, AnkG^32^, TIMP3^32^, ADRA2A^34^ and CRYAB^30,35^. In our in vitro model system, TM cells are small cuboidal and express TM markers such as AQP1, CHI3L1, AnkG and MGP as well as embryonic stem cell markers such as Sox2, Oct4 and Nanog, and neural crest markers such as p75NTR, FOXD3 and Sox10^27^. Although the expression of these markers is gradually reduced in TM cells expanded on 2D Matrigel after serial passage, reseeding them in 3D Matrigel restores TM phenotype and gains the progenitor status confirmed by differentiation potential into corneal endothelial cells, chondrocytes and adipocytes, but not osteocytes or keratocytes, via activation of canonical BMP signaling^27^. Herein, we used this model system to test the hypothesis that a basement membrane matrix with low rigidity plays an active role in maintaining the TM phenotype and withstanding an elevated level of exogenous TGF-β1 through suppression of scarring-prone canonical TGF-β signaling and activation of pFAK-pSrc-pP190RhoGAP-P120RasGAP signaling.

## Results

### 3D Matrigel maintains TM phenotype and TGF-β expression

**Figure 1:**
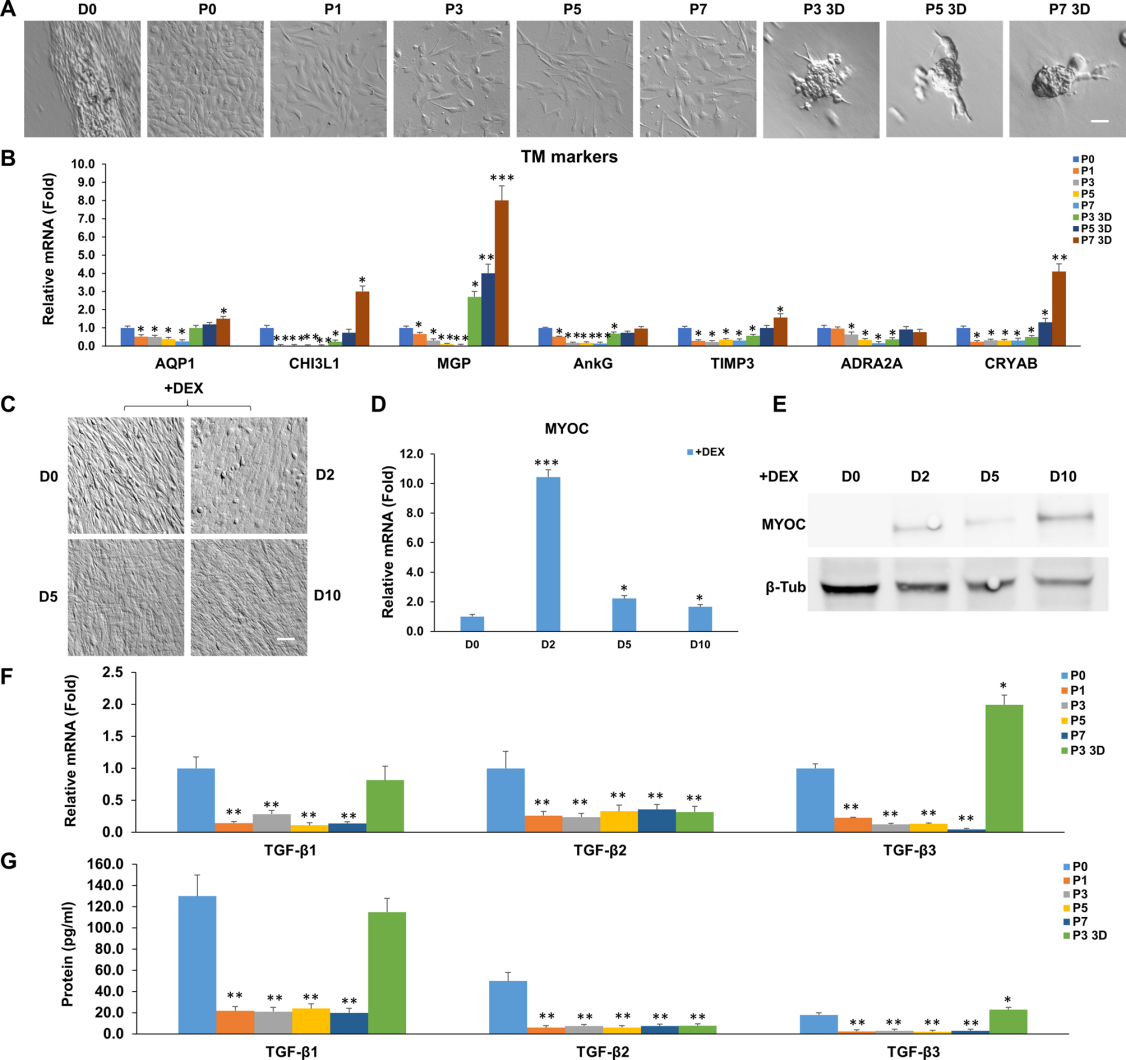
3D Matrigel maintains TM phenotype and TGF-β expression. Freshly isolated TM cells (D0) were serially passaged from P0 to P7 on 2D Matrigel in MESCM + 5% FBS. P2 cells cultured on 2D Matrigel in MESCM + 5% FBS were seeded as P3 in 3D Matrigel for 48 h and passaged to P7 in MESCM + 5% FBS. Cell morphology was demonstrated by phase-contrast microscopy (**A**, scale bar: 100 µm). Folds of transcript expression of TM markers were measured by qRT-PCR using the expression level of P0 TM cells set as 1 (**B**, n = 3, *P < 0.05, **P < 0.01 and ***P < 0.001). Upon confluence, P3 TM cells on 2D Matrigel in MESCM + 5% FBS were treated with 100 nM dexamethasone (DEX) for 10 days. Cell morphology was analyzed by phase-contrast microscopy at Day 0, 2, 5 and 10 after DEX treatment (**C**, scale bar: 100 µm). qRT-PCR was used for quantitation of transcript levels of myocilin (MYOC) at Day 0, 2, 5 and 10 using the expression level by the cells cultured at Day 0 set as 1 (**D**, n = 3, *P < 0.05, **P < 0.01 and ***P < 0.001). Western blotting was used for MYOC protein level at Day 0, 2, 5 and 10 using β-tubulin as the loading control (**E**). In serially passaged cells, folds of transcript expression of TGF-βs were measured by qRT-PCR using the expression level of P0 TM cells set as 1 (**F**, n = 3, *P < 0.05, **P < 0.01 and ***P < 0.001). Levels of TGF-βs proteins in culture media were measured by respective ELISA (**G**, n = 3, *P < 0.05, **P < 0.01 and ***P < 0.001).

Similar to what we have reported^27^, TM cell morphology changed from more cuboidal cells to spindle cells after serial passage on 2D Matrigel in MESCM + 5% FBS from P1 to P7 (Fig. 1A). Also consistent with our report^27^, seeding of P2 TM cells from 2D Matrigel back to 3D Matrigel resulted in cell aggregation, which was maintained after continuous passage in 3D Matrigel to P7 (Fig. 1A). Serial passage on 2D Matrigel significantly diminished the transcript expression of severe different TM markers such as AQP1, CHI3L1, MGP, AnkG, TIMP3, ADRA2A, and CRYAB when compared to that from P0 TM cells (Fig. 1B). However, such a loss could be regained by seeding P2 cells in 3D Matrigel and expression of all seven TM markers except ADRA2A was further upregulated by continuous passage to P7 in 3D Matrigel (Fig. 1B). As reported^36^, the TM cell phenotype is highlighted by significant upregulation of MYOC mRNA and protein by dexamethasone, a TM phenotype not shared by neighboring cells (reviewed in^28,29^). Before DEX treatment, our confluent TM cells were spindle on 2D Matrigel in MESCM + 5% FBS. They became more flatted after DEX treatment (Fig. 1C). Significant upregulation of MYOC mRNA and protein by DEX was noted (Fig. 1D,E). Therefore, these data collectively allowed us to conclude that TM cells expanded in 3D Matrigel maintained the TM cell phenotype. For the first time, we noted that the above TM phenotype was accompanied by expression of TGF-β1, TGF-β2, and TGF-β3. Serial passage on 2D Matrigel resulted in a significant loss of expression of TGF-β1, TGF-β2, and TGF-β3 transcripts (Fig. 1F) and proteins (Fig. 1G). Expression of TGF-β1 and TGF-β3, but not TGF-β2, was restored in 3D Matrigel (Fig. 1F,G).

### 3D Matrigel maintains TM phenotype despite exogenous TGF-β1

**Figure 2:**
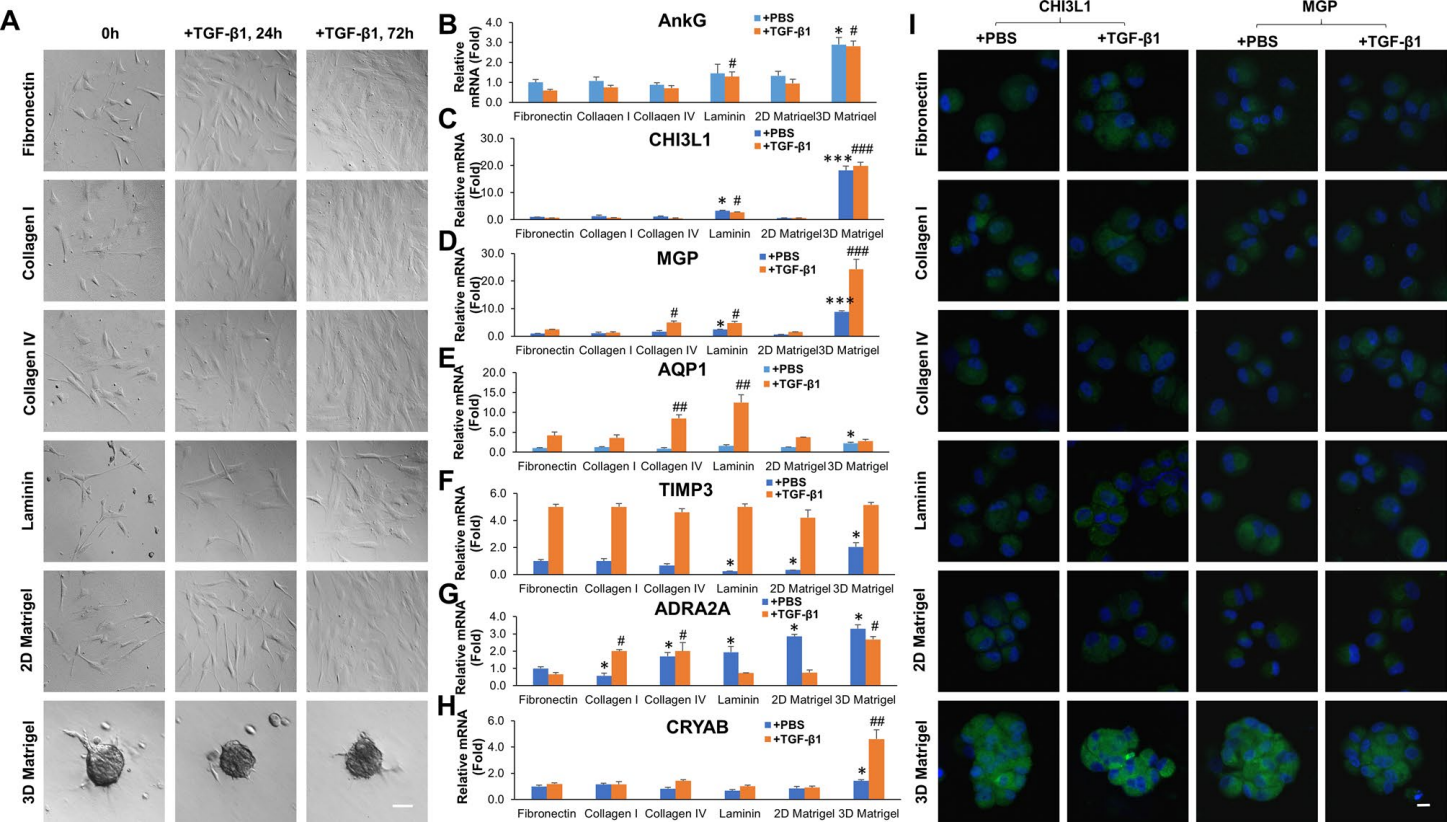
3D Matrigel maintains TM phenotype despite exogenous TGF-β1. P3 cells cultured on 2D Matrigel in MESCM + 5% FBS were seeded on fibronectin, collagen I, collagen IV, laminin, 2D Matrigel or 3D Matrigel for 24 h before being switched to DMEM/F12/ITS for 24 h with or without 10 ng/ml TGF-β1 for another 24 h. The cell morphology was analyzed by phase-contrast microscopy at 0, 24, and 72 h after TGF-β1 treatment (**A**, scale bar: 100 µm). qRT-PCR was used for quantitation of transcript levels of seven TM markers at 24 h using the expression level by cells cultured on fibronectin without (blue) or with (orange) TGF-β1 set as 1, respectively (**B**–**H**, n = 3, *P < 0.05, **P < 0.01 and ***P < 0.001; or ^**#**^P < 0.05, ^**##**^P < 0.01 and ^**###**^P < 0.001). Immunostaining of CHI3L1 and MGP was performed at 24 h (**I**, nuclear counterstained by Hoechst 33,342, scale bar: 20 µm).

To determine whether the aforementioned finding was unique to 3D Matrigel, P3 TM cells cultured on 2D Matrigel were seeded on different substrates including fibronectin, collagen I, collagen IV, laminin, 2D or 3D Matrigel. After 24 h, all TM cells attached and spread well except those on laminin, of which some were round and detached, whereas cells in 3D Matrigel uniquely formed aggregates (Fig. 2A, left column). After switching to a serum-free medium with or without exogenous 10 ng/ml TGF-β1 for 24 h, TM cells on fibronectin, collagen I, collagen IV, laminin or 2D Matrigel became spindle (Fig. 2A, middle column) and enlarged at 72 h after TGF-β1 treatment (Fig. 2A, right column). In contrast, TM cells remained aggregates in 3D Matrigel (Fig. 2A, bottom panel). These results strongly suggested that the TM cell morphology was uniquely maintained in 3D Matrigel despite being challenged by exogenous TGF-β1. Compared to the control cultured on fibronectin, the transcript expression of AnkG, CHI3L1, MGP, AQP1 TIMP3, ADRA2A and CRYAB was significantly upregulated in TM cells cultured in 3D Matrigel without exogenous TGF-β1 (Fig. 2B–H, blue bars) while such upregulated transcript levels were maintained or further upregulated with exogenous TGF-β1 (Fig. 2B–H, orange bars). Among all other substates, laminin upregulated transcript expression of CHI3L1, MGP and ADRA2A; collagen IV and 2D Matrigel upregulated transcript expression of ADRA2A without TGF-β1 (Fig. 2B–H, blue bars). After TGF-β1 treatment, laminin upregulated transcript expression of AnkG, CHI3L1, MGP and AQP1, collagen IV upregulated transcript expression of MGP, AQP1 and ADRA2A, while collagen I only upregulated transcript expression of ADRA2A (Fig. 2B–H, orange bars). Immunostaining disclosed strong cytoplasmic staining of CHI3L1 and MGP in cells in 3D Matrigel with or without TGF-β1 but weak or no expression in cells on all other substrates (Fig. 2I). Therefore, 3D Matrigel uniquely maintained TM phenotype despite being challenged by exogenous TGF-β1.

### TGF-β1 and TGF-β2 dose-dependently upregulate αSMA expression and stress fibers on fibronectin but not in 3D Matrigel

**Figure 3:**
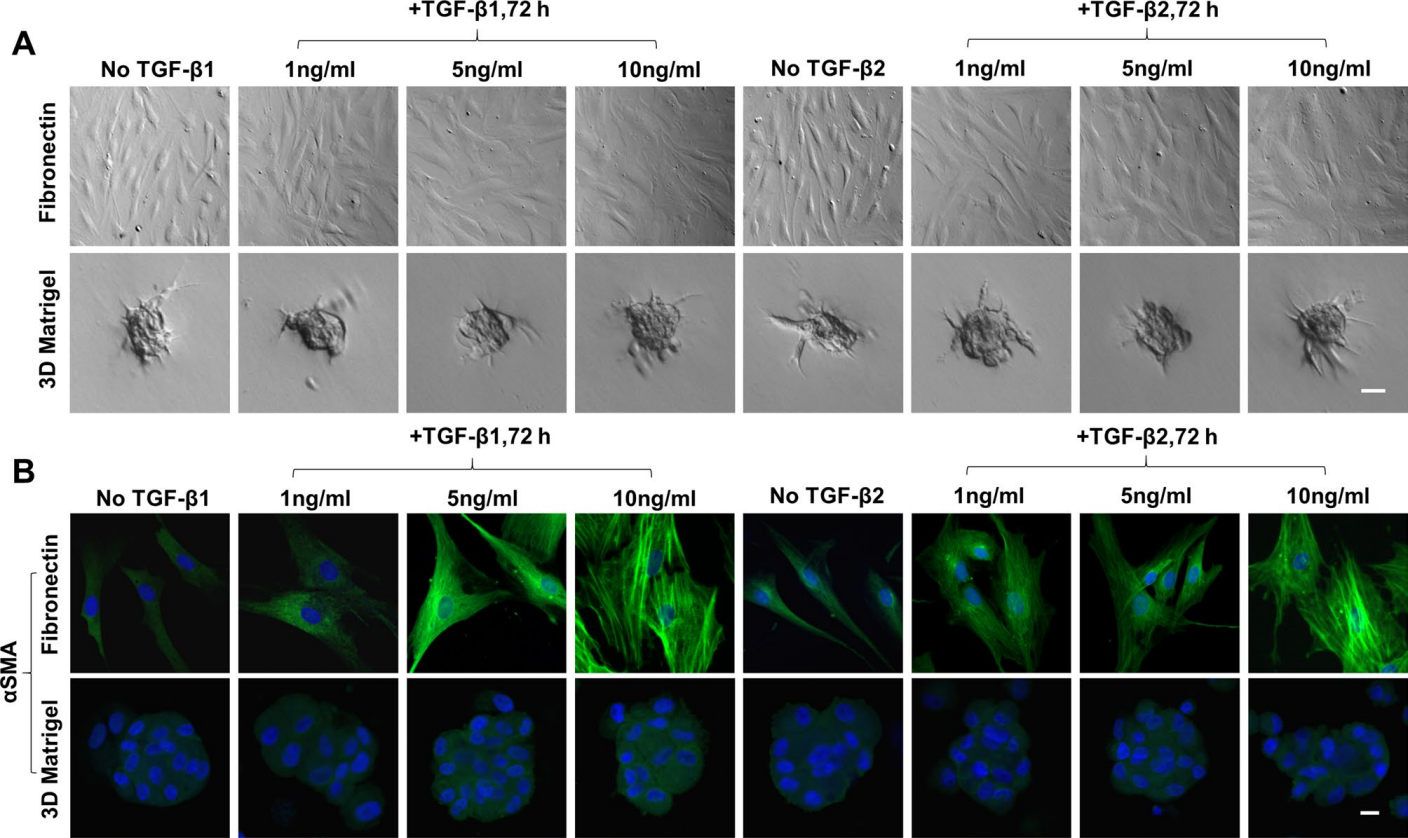
TGF-β1 and TGF-β2 dose-dependently upregulate αSMA Expression and Stress Fibers on Fibronectin but not in 3D Matrigel. P3 cells cultured on 2D Matrigel in MESCM + 5% FBS were seeded on fibronectin or in 3D Matrigel for 24 h before being switched to DMEM/F12/ITS for 24 h and then with 0, 1, 5, or 10 ng/ml TGF-β1 or TGF-β2 for another 72 h. The cell morphology was analyzed by phase-contrast microscopy at 72 h after TGF-β treatment (**A**, scale bar: 100 µm). Immunostaining of αSMA was performed at 72 h (**B**, nuclear counterstained by Hoechst 33342, scale bar: 20 µm).

To further demonstrate the aforementioned difference between 3D Matrigel and other substrates in the response to exogenous TGF-β, we performed a dose–response experiment by seeding P3 TM cells on FN or in 3D Matrigel with or without 1 to 10 ng/ml of TGF-β1 or TGF-β2 for 72 h. As expected, TM cells on fibronectin were enlarged after TGF-β1/2 treatment in a dose-dependent manner while those maintained aggregates in 3D Matrigel (Fig. 3A). On fibronectin, there was an increasing positive immunofluorescence staining of αSMA in TM cells in a dose-dependent manner after being treated with TGF-β1 or TGF-β2 and stress fibers became apparent after treated with 10 ng/ml of TGF-β1 or TGF-β2 (Fig. 3B, top panel). In contrast, TM cells cultured in 3D Matrigel did not express αSMA or stress fibers (Fig. 3B, bottom panel), confirming that 3D Matrigel was unique in withstanding the challenge of exogenous TGF-β1 and TGF-β2.

### 3D Matrigel suppresses myofibroblast differentiation by downregulating smad2/3 signaling

**Figure 4:**
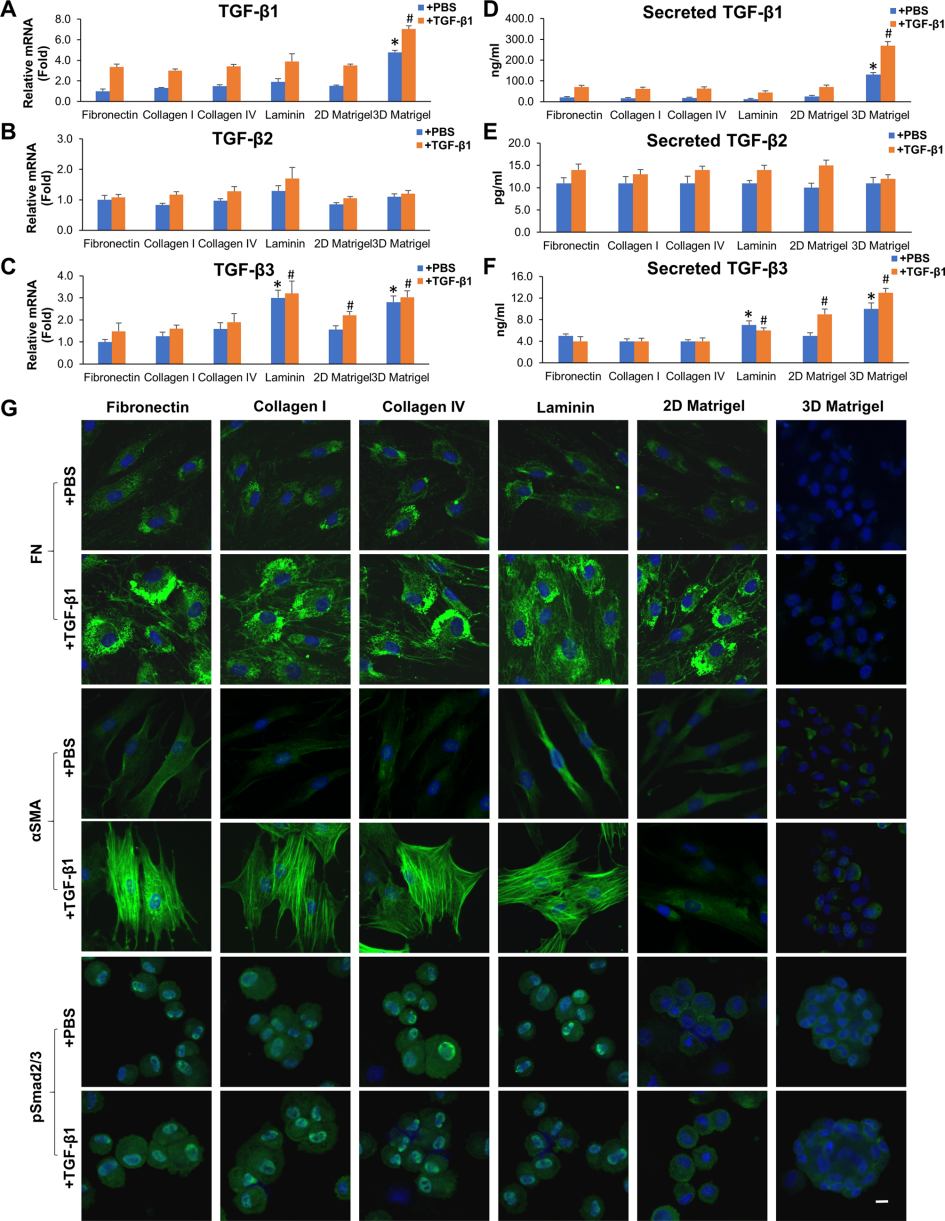
3D Matrigel suppresses myofibroblast differentiation by downregulating Smad2/3 Signaling. P3 cells cultured on 2D Matrigel in MESCM + 5% FBS were seeded on fibronectin, collagen I, collagen IV, laminin, 2D Matrigel, or 3D Matrigel for 24 h before being switched to DMEM/F12/ITS for 24 h with or without 10 ng/ml TGF-β1 for another 24 h. Expression levels of TGF-βs transcripts were measured by qRT-PCR at 24 h using the level expressed by cells cultured on fibronectin without (blue) or with (orange) TGF-β1 set as 1, respectively (**A**–**C**, n = 3, *P < 0.05 and ^**#**^P < 0.05). Expression levels of TGF-βs proteins in culture media measured by ELISA were compared to cells cultured on fibronectin without (blue) or with (orange) (**D**–**F**, n = 3, *P < 0.05 or ^**#**^P < 0.05). TM cells were subjected to immunostaining of pSmad2/3 at 24 h and fibronectin and αSMA at 72 h (**G**, nuclear counterstained by Hoechst 33342, scale bar: 20 µm).

Because both TGF-β1 and TGF-β2 exhibited comparable dose-dependent responses in promoting myofibroblast differentiation as evidenced by expression of αSMA and stress fibers (Fig. 3), we thus selected 10 ng/ml of TGF-β1 for the remaining experiments. Compared to TM cells cultured on fibronectin, addition of TGF-β1 further upregulated expression of TGF-β1 and TGF-β3 transcripts (Fig. 4A–C) and proteins (Fig. 4D–F) and such upregulation was more prominent in TM cells cultured in 3D Matrigel. Among all other substrates, laminin and 2D Matrigel also upregulated expression of TGF-β3 transcript and protein (Fig. 4C,F). Expression of TGF-β2 transcript and protein was not affected by exogenous TGF-β1 on all substrates (Fig. 4B,E). Interestingly, cytoplasmic staining of fibronectin, αSMA, and nuclear pSmad2/3 staining were pronounced in cells cultured on fibronectin, collagen I, collagen IV and laminin following addition of TGF-β1 (Fig. 4G). TM cells showed mildly increased cytoplasmic staining of fibronectin, weak staining of αSMA, but negative nuclear pSmad2/3 staining when cultured on 2D Matrigel (Fig. 4G, fifth column). In contrast, cell aggregates in 3D Matrigel exhibited no staining of fibronectin and αSMA and cytoplasmic staining of pSmad2/3 (Fig. 4G, right column). Collectively, these results strongly suggested that canonical TGF-β signaling was activated in TM cells cultured on fibronectin, collagen I, collagen IV and laminin after addition of TGF-β1 and such signaling was accompanied by deposition of fibronectin and myofibroblast differentiation. In contrast, canonical TGF-β signaling was not activated in TM cells cultured on 2D or in 3D Matrigel despite addition of TGF-β1 and was not accompanied by fibronectin deposition and myofibroblast differentiation.

### 3D Matrigel uniquely withstands RhoA activation by pFAK-pSrc-pP190RhoGAP-P120RasGAP signaling

**Figure 5:**
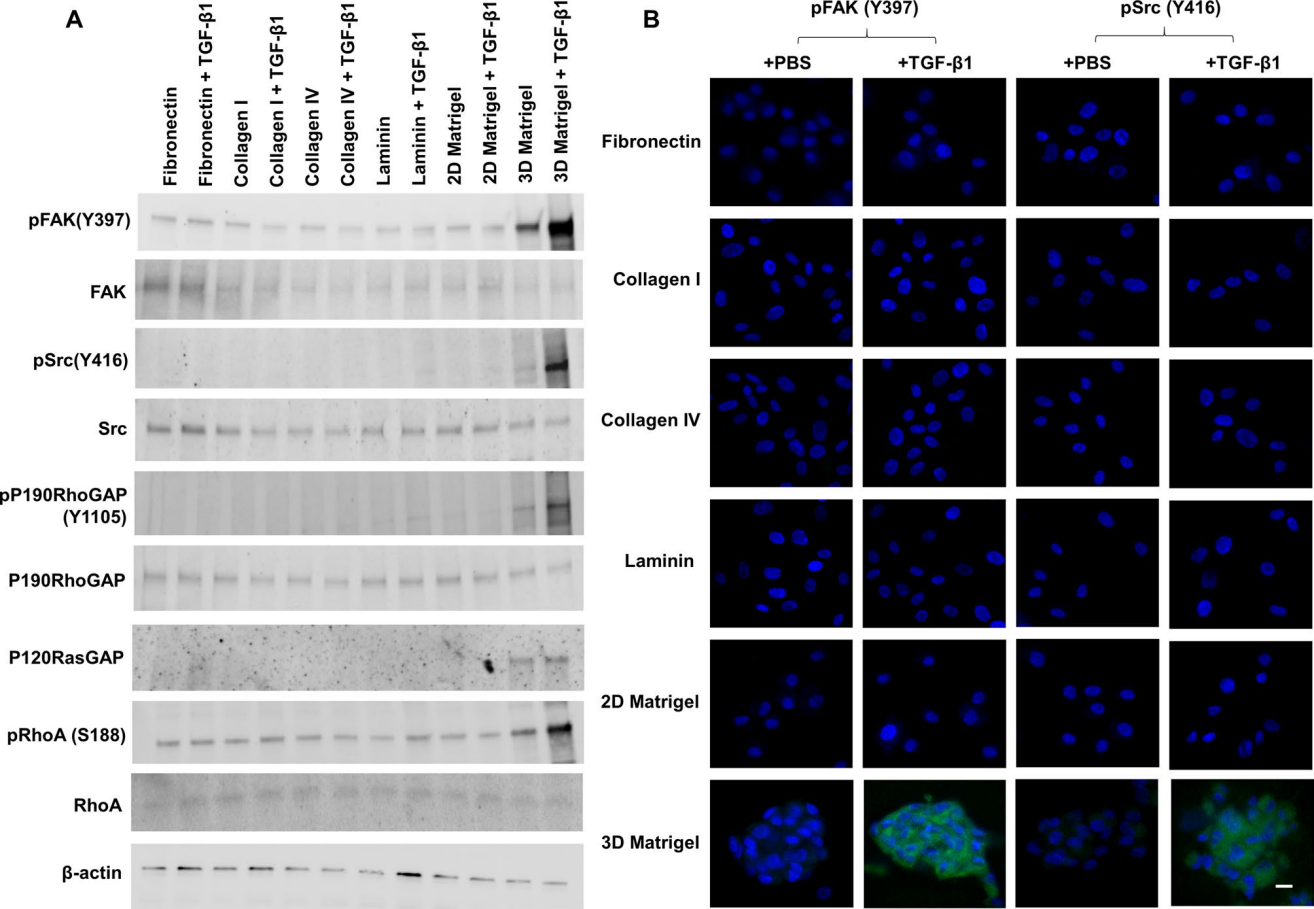
3D Matrigel uniquely withstands RhoA activation by pFAK-pSrc-pP190RhoGAP-P120RasGAP signaling. P3 cells cultured on 2D Matrigel in MESCM + 5% FBS before being switched to DMEM/F12/ITS for 24 h. After serum starvation in DMEM/F12/ITS, cells reseeded on fibronectin, collagen I and IV, laminin, 2D Matrigel and 3D Matrigel with or without 10 ng/ml TGF-β1 for 15 min were harvested for Western blotting of pFAK(Y397), FAK, pSrc(Y416), Src, pP190RhoGAP(Y1105), P190RhoGAP, P120RasGAP, pRhoA(S188) and RhoA using β-actin as the loading control (**A**) and immunostaining of pFAK(Y397) and pSrc(Y416) (**B**, nuclear counterstained by Hoechst 33342, scale bar: 20 µm).

Because focal adhesion kinase (FAK) can be activated by TGF-β1 at sites of integrin/matrix engagement, we wondered whether FAK signaling might be involved as one non-canonical TGF-β signaling. To test this hypothesis, P3 cells cultured on 2D Matrigel in MESCM + 5% FBS were switched to DMEM/F12/ITS for 24 h and passaged on fibronectin, collagen I and IV, laminin, 2D Matrigel or 3D Matrigel with or without 10 ng/ml TGF-β1 for 15 min. The result showed that the pFAK (Y397) band was promoted by 3D Matrigel and further promoted by 3D Matrigel with additional TGF-β1 when compared to other substrates (Fig. 5A). Accompanied by activation of pFAK (Y397) in 3D Matrigel was marked upregulation of pSrc (Y416), pP190RhoGAP (Y1105), and P120RasGAP (Fig. 5A). Importantly, upregulation of pFAK (Y397), pSrc(Y416), pP190RhoGAP(Y1105), and P120RasGAP was accompanied by upregulation of pRhoA (S188), which is indicative of inactivation of RhoA^37,38^. Immunostaining confirmed that cytoplasmic staining of pFAK (Y397) and pSrc (Y416) was promoted in TM aggregates in 3D Matrigel in the presence of TGF-β1 (Fig. 5B). Taken together, these results indicated activation of pFAK-pSrc-pP190RhoGAP-P120RasGAP signaling to inhibit RhoA by TGF-β1 in TM cells seeded in 3D Matrigel.

### FAK inhibitor 14 downregulates TM markers, upregulates expression of matrix markers and αSMA, and activates canonical TGF-β signaling in 3D Matrigel

**Figure 6:**
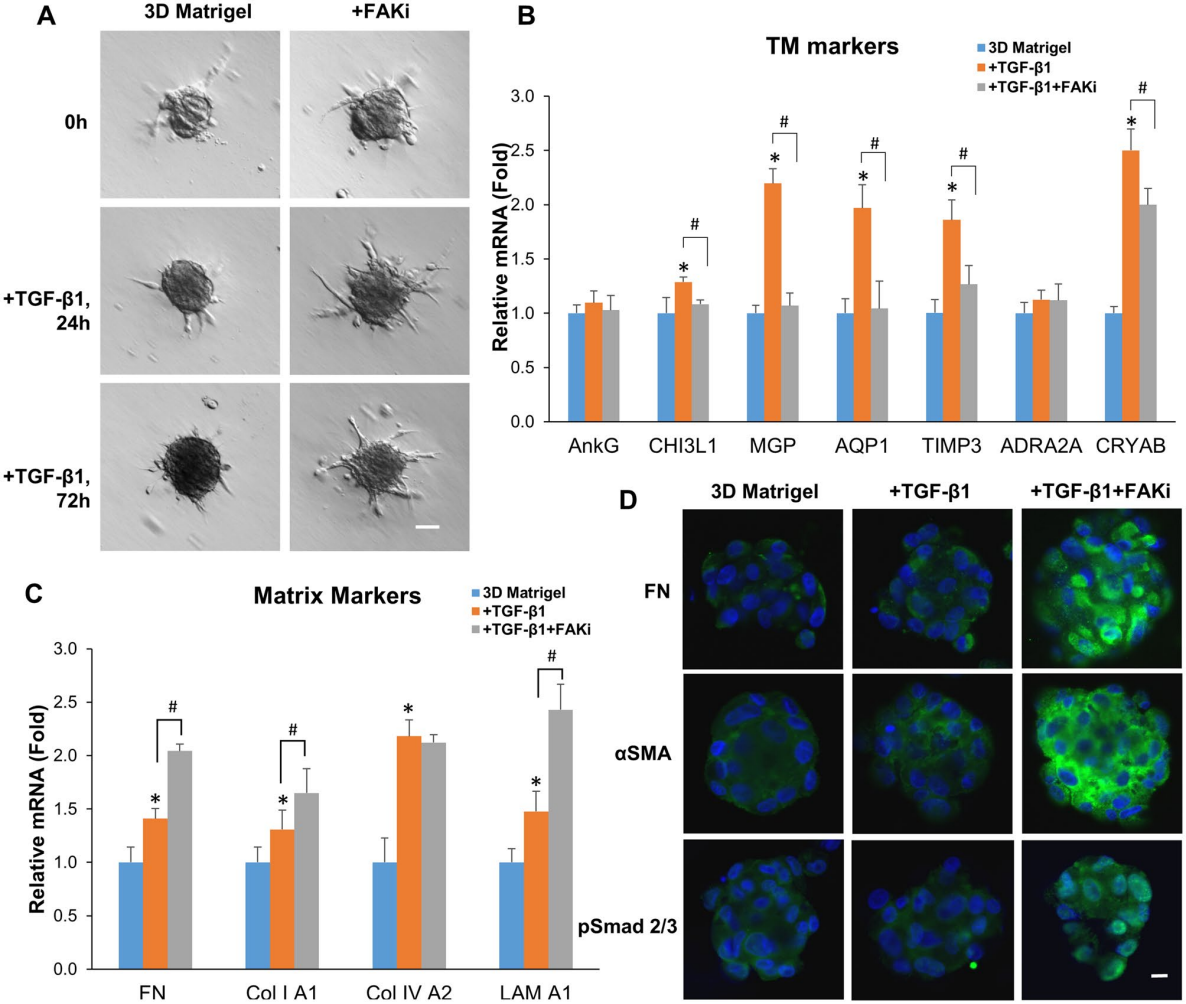
FAK inhibitor 14 downregulates TM markers, upregulates expression of matrix markers and αSMA, and activates canonical TGF-β signaling in 3D Matrigel. P3 cells cultured on 2D Matrigel in MESCM + 5% FBS were seeded in 3D Matrigel with or without FAK inhibitor 14 for 24 h and then switched to DMEM/F12/ITS for 24 h with 10 ng/ml TGF-β1. The cell morphology was analyzed by phase-contrast microscopy at 0, 24, and 72 h after TGF-β1 treatment (**A**, scale bar: 100 µm). At 24 h, cells were subjected to qRT-PCR for quantitating the fold transcription expression of TM markers and matrix markers using cells cultured in 3D Matrigel set as 1 without (blue) or with (orange) TGF-β1 set as 1 (**B** and **C**, n = 3, *P < 0.05 and ^**#**^P < 0.05). Cells were subjected to immunostaining of pSmad2/3 at 24 h and αSMA and fibronectin at 72 h (**D**, nuclear counterstained by Hoechst 33,342, scale bar: 20 µm).

To further evaluate the causal relationship mediated by the FAK-Src signaling, P3 cells cultured on 2D Matrigel in MESCM + 5% FBS were seeded in 3D Matrigel and then were added with 3 μM of FAK inhibitor 14^39^ for 24 h before being switched to DMEM/F12/ITS with 10 ng/ml TGF-β1 for 24 h. Addition of FAK inhibitor 14 did not change formed aggregates after addition of TGF-β1 (Fig. 6A). However, the upregulated transcript expression of CHI3L1, MGP, AQP1, TIMP3 and CRYAB by TGF-β1 in 3D Matrigel was significantly downregulated by FAK inhibitor 14 (Fig. 6B). The upregulated transcript expression of fibronectin, collagen I A1, and Laminin A1 by TGF-β1 in 3D Matrigel was further upregulated by FAK inhibitor 14 (Fig. 6C). Furthermore, addition of FAK inhibitor 14 promoted cytoplasmic staining of fibronectin and αSMA as well as nuclear staining of pSmad2/3 in TM aggregates cultured in 3D Matrigel in the presence of TGF-β1 (Fig. 6D). Therefore, blockade of FAK by its inhibitor 14 resulted in downregulation of TM markers, upregulation of matrix markers including fibronectin and αSMA, and activation of canonical Smad2/3-mediated TGF-β signaling in TM cells cultured in 3D Matrigel in the presence of TGF-β1.

## Discussion

**Figure 7:**
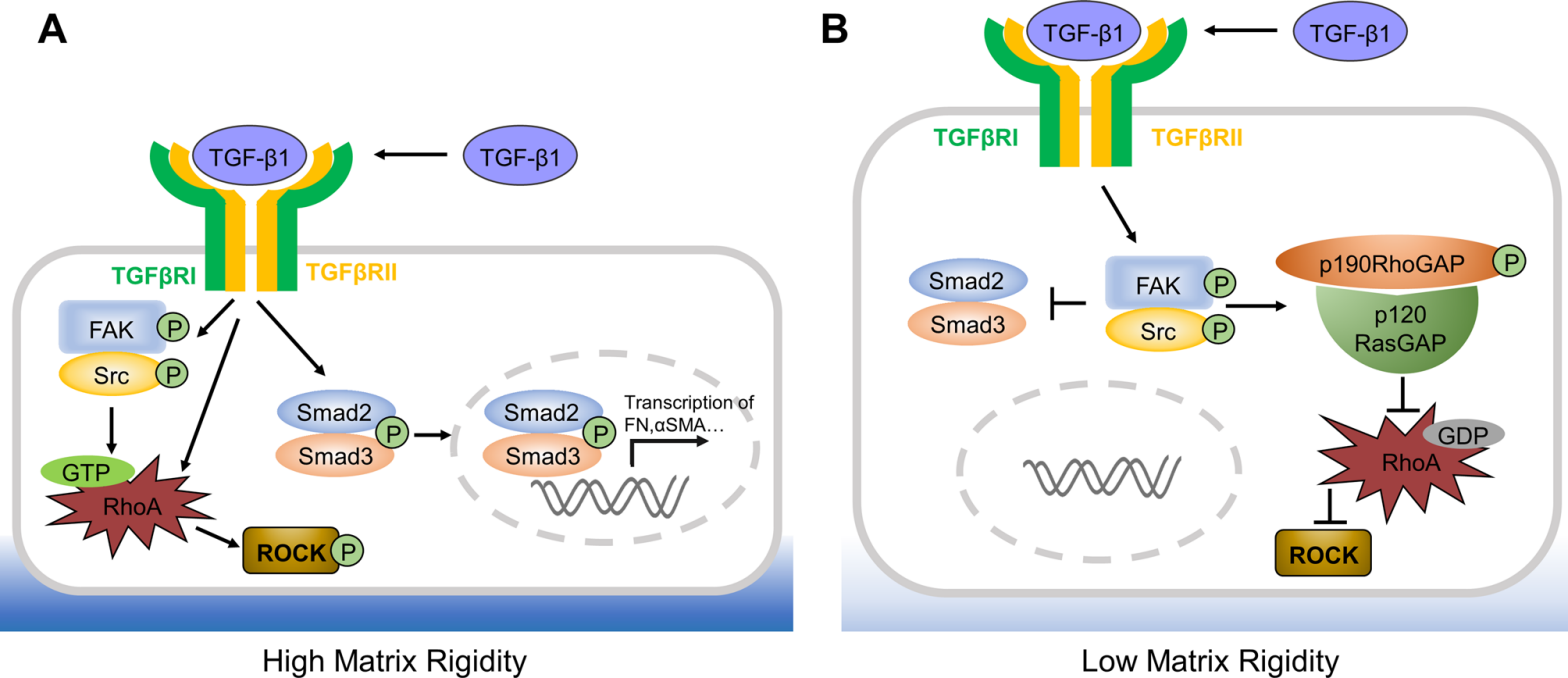
Matrix modulation of TM phenotype via canonical or non-canonical TGF-β signaling. (**A**) On substrates with high matrix rigidity such as fibronectin, collagen I, collagen IV or laminin, canonical TGF-β signaling mediated by Smad2/3 and non-canonical signaling such as RhoA-ROCK are activated in TM cells resulting in abnormal phenotype with high cellular contractility and abnormal ECM deposition, mimicking the glaucoma state. (**B**) In matrix with low rigidity such as 3D Matrigel, activation of both canonical TGF-β signaling and RhoA-ROCK signaling is suppressed by pFAK-pSrc-pP190RhoGAP-P120RasGAP signaling even under high concentrations of TGF-β1, mimicking the normal state.

TM cells lie on beams of ECM, which consists of fibronectin, collagen IV, laminin and other substrates^5,40,41^. Glaucoma TM ECM is characterized by increased deposition of matrix components such as fibronectin, collagens type I, III, V, VI, XI, XII and XIV^42,43^. Following the binding of TGF-βs with TGF-βRI and TGF-βRII, the canonical signaling pathway leads to nuclear translocation of pSmad2/3 that triggers transcriptional activation of many downstream genes including fibronectin and αSMA that are found in pathological fibrosis in many tissues^44,45^ (also depicted in Fig. 7A). Besides canonical Smad-mediated signaling, TGF-β also elicits non-canonical signaling such as RhoA-ROCK signaling leading to stress fiber formation and cytoskeletal reorganization^46^ (depicted in Fig. 7A). Herein, our study showed that myofibroblast differentiation, fibronectin deposition and nuclear staining of pSmad2/3 were accompanied by the loss of expression of TM markers when TGF-β1 was added to TM cells cultured on fibronectin, collagen I, collagen IV or laminin (Fig. 4). Therefore, we conclude that an elevated TGF-β level will elicit canonical TGF-β signaling when TM cells are exposed to these scar-prone matrices, mimicking the pathological state of glaucoma as reported by others by culturing TM cells on plastic with or without laminin^47–49^, fibronectin or collagens^50^. Because altered ECM may act as a fibrogenic niche leading to tissue fibrosis^51^, we surmise that altered ECM plays a “pathological” role in perpetuating TM cells to respond to TGF-β from AH to adopt an abnormal phenotype with increased cellular contractility and deposition of altered ECM^23,24,52^.

Our study further validated that cells expanded in 2D Matrigel and reseeded in 3D Matrigel were indeed TM cells as evidenced by expression and further upregulation of all seven putative TM markers, i.e., MGP, CHI3L1, AQP1, AnkG, TIMP3, ADRA2A and CRYAB in cell aggregates after continuous passage (Fig. 1A,B). Furthermore, these TM cells also exhibited another TM feature, i.e., significant upregulation of MYOC by DEX (Fig. 1C–E) as reported^27^. Besides expression of these putative TM markers, our study also demonstrated for the first time that these TM cells expressed all three isoforms of TGF-β (Fig. 1), suggesting that expression of TGF-βs is another key feature of the normal TM phenotype. The expression of TGF-β3 by TM cells is particularly intriguing as TGF-β3 differs from TGF-β1 and TGF-β2 in exerting an anti-scarring action^53–55^ and promoting deposition of normal ECM by corneal stromal cells^56,57^. Further studies are needed to determine the role of TM cells in producing TGF-βs present in AH and whether expression of TGF-β3 is an important feature to maintain TM phenotype.

Unlike other substrates, TM cells cultured in 3D Matrigel maintained the normal but not “pathological” phenotype even in the presence of a high concentration of TGF-β1, which was comparable to TGF-β2 as judged by a dose–response relationship (Fig. 3) by suppression of canonical Smad-mediated TGF-β signaling (Fig. 4, also depicted in Fig. 7B). Matrigel, a basement membrane matrix extracted from Engelbreth-Holm-Swarm mouse sarcoma consisting of laminin, collagen IV, entactin/nidogen, heparan sulfate proteoglycans and growth factors such as TGF-β and EGF^58^, has matrix stiffness (rigidity) of 50 Pa (3D, 50% Matrigel)^59^, which is significantly lower than that of collagen I (more than 6 kPa)^60,61^, fibronectin (up to 40 kPa)^62^, and laminin (up to 250 Pa)^63^. Besides matrix rigidity, our study showed that one action mechanism leading to the suppression of canonical Smad-mediated TGF-β signaling in 3D Matrigel could be activation of FAK-mediated signaling (Fig. 5). Addition of a small FAK inhibitor resulted in pathological manifestation characterized by activation of canonical Smad-mediated TGF-β signaling, downregulation of TM markers, and upregulation of αSMA and matrix marker in TM cells in 3D Matrigel (Fig. 6). FAK is a non-receptor cytoplasmic protein tyrosine kinase and activated when cells bind to ECM proteins through integrin receptors^64–68^ during cell adhesion on different substrates in cooperation with various growth factors including TGF-β^69–75^.

FAK can be activate by TGF-β1 at sites of integrin/matrix engagement^76^. TGF-β1 induces FAK activation in a time and dose dependent manner that precedes expression of αSMA and collagen deposition in liver fibrosis^77^. Activation of FAK is through phosphorylation on Y397 as a high-affinity binding site for the SH2 domain of Src family kinases and leads to the recruitment and activation of Src via Y416^78,79^. The downstream signaling of pFAK(Y397)-pSrc(Y416) may lead to αSMA cytoskeleton reorganization, profibrotic gene expression and matrix deposition through MEKK1-JNK^75^, PI3K-Hippo^80^, and RhoA-ROCK^76,81^. Therefore, TGF-β1 may activate RhoA-ROCK through non-canonical signaling with or without involvement of FAK (Fig. 7). However, FAK serves as a “switch” for multiple signaling outputs (for reviews see^64,82,83^). For example, FAK is a critical component of a pathway leading to signals that either positively or negatively modulate the assembly and breakdown of adhesions at the leading and/or trailing edges of migrating cells^84^. The downstream signaling of pFAK(Y397)-pSrc(Y416) for TM cells in 3D Matrigel uniquely activated a complex of pP190RhoGAP (Y1105) and P120RasGAP (Fig. 5). This scenario has been reported in C3H10T1/2 murine fibroblasts^85^, chicken embryo fibroblasts, and vascular endothelial cells leading to cytoskeletal rearrangement via disassembly of actin stress fibers^85,86^ to facilitate cell migration^85,87,88^, cell–cell contact^89^, and endothelial cell polarity and barrier function^90,91^. In 3D Matrigel, activation of downstream p190RhoGAP and p120RasGAP complex resulted in inhibition of RhoA activity as evidenced by S188 phosphorylation (Fig. 5), a hallmark of suppressed RhoA signaling^37,38^. Therefore, we conclude that activation of pFAK-pSrc-pP190RhoGAP-P120RasGAP signaling in 3D Matrigel uniquely suppresses both RhoA and TGF-β Smad-mediated signaling to maintain TM morphology and phenotype and prevent differentiation into contractility-prone myofibroblasts even under exogenous challenge of TGF-β1 (Fig. 7B). Further studies are needed to determine whether in vivo TM ECM possesses the biochemical and physical properties similar to 3D Matrigel and plays an active role in maintaining normal TM phenotype and withstanding the challenge of an elevated level of TGF-β2 in AH.

## Methods

### Materials

Materials used for culture and relevant experiments of TM cells were detailed in Supplementary Table S1. Primers for quantitative Real-time PCR (qRT-PCR) were listed in Supplementary Table S2. Antibodies used for immunostaining and Western blotting were detailed in Supplementary Table S3. ELISA kits were shown in Supplementary Table S4.

### Isolation, expansion and treatment of TM Cells on 2D Matrigel

Human donor corneas from deidentified cadaver sources were provided by Florida tissue banks and handled in a HIPAA-compliant manner in accordance with the tenets of the Declaration of Helsinki. This study was reviewed and approved by Tissue Tech Inc, Miami, Florida, USA. TM cells were isolated and expanded as previously reported^27^. In brief, human corneoscleral rims stored at 4 °C for less than 7 days were obtained from Florida Lions Eye Bank (Miami, FL). Under a dissecting microscope, the corneoscleral rim was stripped off the iris by forceps and was cut through the inner edge of Schwalbe’s line delineated by pigmentation as reported by Keller et al.^29^. After rinsing with MESCM, the Descemet’s membrane was stripped off. The remaining TM tissue was removed and digested with 2 mg/ml collagenase A at 37 °C for 16 h (overnight) in MESCM, which is made of Dulbecco’s Modified Eagle’s Medium (DMEM)/F-12 nutrient mixture (F-12) (1:1) with 10% knockout serum, 4 ng/ml bFGF, 10 ng/ml LIF, 5 ng/ml sodium selenite supplement, 5 mg/ml transferrin, 5 mg/ml insulin, 1.25 μg/ml amphotericin B, 50 μg/ml gentamicin and 5% FBS^92^. The resultant cells were washed once in MESCM + 5% FBS and seeded at a density of 2 × 10^3^ per cm^2^ for expansion on plastic coated with 2D Matrigel (5% diluted Matrigel) after 1 h incubation at 37 °C in MESCM + 5% FBS. Upon 80–90% confluence, TM cells were passaged serially at a density of 5 × 10^3^ per cm^2^ in MESCM + 5% FBS. Passage 3 (P3) TM cells were cultured until confluence and were maintained another 5 days to obtain stable monolayers before being used for experiments. These cells were treated with or without 100 nM dexamethasone (DEX) for up to 10 days to assess expression of myocillin^36^.

### Seeding TM cells on different substrates with or without TGF-β

P3 TM cells expanded on 2D Matrigel in MESCM + 5% FBS were digested by 0.25% Trypsin/EDTA upon 70–80% confluence and seeded at the density of 1 × 10^4^ cells per cm^2^ on plastic coated with 2D Matrigel or 3D Matrigel (50% diluted Matrigel) after 1 h incubation at 37 °C, or plastic coated with fibronectin (0.2 ml/cm^2^), collagen I (5 µg/cm^2^), collagen IV (10 µg/cm^2^) or laminin (5 µg/cm^2^) after overnight incubation at 4 °C in MESCM + 5%FBS. After serum starvation for 24 h, 1–10 ng/ml TGF-β1 or TGF-β2 was added. In addition, FAK inhibitor 14^39^ was also added at the final concentration of 3 μM for 72 h in in MESCM + 5% FBS in 3D Matrigel.

### Quantitative real-time PCR (qRT-PCR)

These steps are similar to our previous report^27^. In brief, total RNAs were extracted from TM cells in different groups using a RNeasy kit and reverse-transcribed using a High Capacity Reverse Transcription kit. cDNA of each group was amplified by qRT-PCR using specific primer–probe mixtures and DNA polymerase in a real-time PCR system (QuantStudio; ThermoFisher). The PCR profile consisted of 10 min of initial activation at 95 °C, followed by 40 cycles of 15 s denaturation at 95 °C, and 1 min annealing and extension at 60 °C.

### Western blotting

Cell lysates were collected using RIPA buffer and loaded in 4–15% (wt/vol) gradient acrylamide gels for Western blotting. In brief, the protein extracts were transferred to a nitrocellulose membrane, which was then blocked with 2% (w/v) BSA in TBST (prepared with 50 mM Tris- HCl, pH 7.5, 150 mM NaCl, and 0.05% [vol/vol] Tween-20), followed by incubation with specific primary antibodies against MYOC, pFAK (Y397), FAK, pSrc (Y416), Src, pP190RhoGAP (Y1105), P190RhoGAP, P120RasGAP and pRhoA (S188) and their corresponding secondary antibodies using β-tubulin or β-actin as the loading control. Immunoreactive proteins were analyzed with Western Lighting Chemiluminescence (PerkinElmer).

### Immunofluorescence staining

The staining procedures are similar to our previous report^27^. In brief, after being treated with or without TGF-β1 for 15 min, 24 h or 72 h in different groups, single TM cells after trypsin/EDTA digestion or 3D clusters were harvested and prepared by cytospin at 1000 rpm for 4 min (StatSpin, Inc., Norwood, MA). The cytospin slides or cells cultured on cover slips were fixed with 4% paraformaldehyde for 15 min, permeabilized with 0.2% Triton X-100 in PBS for 30 min, and blocked with 2% BSA in PBS for 1 h before being incubated with specific primary antibodies for 16 h (overnight) at 4 °C. After washing with PBS, samples were incubated with respective secondary antibodies for 1 h at room temperature. After another wash with PBS, the nucleus was counterstained with Hoechst 33342 before being analyzed with confocal microscope (LSM700; Carl Zeiss, Thornhood, NY).

### Enzyme-linked immunosorbent assay (ELISA)

The culture medium from different groups treated with or without TGF-β1 was collected after centrifuge at 300×*g* for 5 min and subjected to the measurement of TGF-β1, TGF-β2 and TGF-β3 proteins by ELISA using their specific kit. For TGF-βs, the medium was collected 24 h after addition of exogenous TGF-β1. The medium was then added to the plate precoated with capture antibody and incubated 2 h at room temperature. After washing, detection antibody was added and incubated for another 2 h at room temperature. After washing, Streptavidin-HRP was added for 20 min. Subsequently, substrate solution was added for 20 min after another wash. After addition of the stop solution, the plate was read by Microplate Reader (Molecular Device SpectraMax M5).

### Statistical analysis

Unpaired t-test (independent sample t-test) was used for comparing the differences between two groups. One-way analysis of variance (ANOVA) followed by Tukey’s HSD post hoc test was used for comparing differences among 3 or more groups. p < 0.05 was considered statistically significant.

## Supplementary Information

Supplementary Information
