# Supplementary material for: Suppression of TGF-β1 signaling by Matrigel via FAK signaling in cultured human trabecular meshwork cells
Source: Sci Rep. 2021 Apr 1;11:7319. doi: 10.1038/s41598-021-86591-7 (PMC8016910; doi:10.1038/s41598-021-86591-7)
Supplement: Supplementary file 1 — Supplementary Information [file 41598_2021_86591_MOESM1_ESM.pdf]

Suppression of TGF-β1 Signaling by Matrigel via FAK Signaling in Cultured Human Trabecular Meshwork Cells

Yuan Zhang, Scheffer C. G. Tseng and Ying-Ting Zhu[#]

Research and Development Department, Tissue Tech, Inc., Miami, FL, USA

Short Running Head: Suppression of TGF-β1 Signaling in Human Trabecular Meshwork Cells

Key Words: Basement membrane, trabecular meshwork, TGF-β, 3D Matrigel, FAK, Src, P190RhoGAP, P120RasGAP, RhoA

[#]**Author for Correspondence**: Ying-Ting Zhu, Ph.D., R&D Department, TissueTech, Inc., 7235 Corporate Center Drive, Suite B, Miami, Florida, 33126. Telephone: (786) 456-7632; Fax: (305) 274-1297; E-mail: yzhu@tissuetechinc.com

Supplementary Table S1. Materials Used for Cell Culturing and Following Experiments

Materials	Sources	Catalog number	Concentration/ Volume
Amphotericin B	Invitrogen, Grand Island, NY	15290026	50 µg/ml
Collagenase A	Roche, Indianapolis, IN	10103586001	2 mg/ml
Collagen I	Corning	354236	5 µg/cm^2
Collagen IV	Sigma-Aldrich,Inc	C5533	10 µg/cm^2
Cell Transformation Assay Kit	Abcam	ab235698	N/A
Dexamethasone	Sigma	D4902-100MG	100nM
Dulbecco's modified Eagle's Medium (DMEM)	Invitrogen, Grand Island, NY	21063029	DMEM/F-12 (1:1)
Dispase II	Life Technology	17105-041	10 mg/ml
FAK Inhibitor 14	Sigma	SML0837	3µM
F-12 nutrient mixture (F-12)	Invitrogen, Grand Island, NY	31765035	DMEM/F-12 (1:1)
Fetal Bovine Serum (FBS)	Invitrogen, Grand Island, NY	10082147	5%
Fibroblast growth factor 2(FGF-2)	Sigma-Aldrich,Inc	SRP4037	10 ng/ml
Fibronectin coating mix	AthenaES	0407	0.2 ml/cm^2
Gentamicin	Invitrogen, Grand Island, NY	15710072	1.25 µg/ml
Human Fibroblast Growth Factor-Basic	Invitrogen, Grand Island, NY	RFGFB50	4 ng/ml
High Capacity Reverse Transcription kit	ThermoFisher	4368813	N/A
Insulin-Transferrin-sodium selenite media supplement	Roche, Indianapolis, IN	10394000	5 µg/ml insulin, 5 µg/ml Transferrin, 5 ng/ml sodium selenite
Knockout Serum Replacement	Invitrogen, Grand Island, NY	10828010	10%
Laminin	Corning	354232	5 µg/cm^2
Leukemia inhibitory factor (LIF)	Sigma-Aldrich,Inc	L9545	10 ng/ml
Matrigel™ Basement Membrane Matrix	Corning	354230	50% or 5%

TGF-β1	PeproTech,Inc	AF-100-21C	10 ng/ml
TGF-β2	PeproTech,Inc	100-35	10 ng/ml
Trypsin and EDTA (T/E)	Invitrogen, Grand Island, NY	25300054	0.05% and 1 mM

Supplementary Table S2. Assay ID Used for Real-time PCR

Gene Name	Vender	Assay ID (Taqman Expression Assay)
ADRA2A	Life Technology	Hs01099503_s1
Col I A1	Life Technology	Hs00164004_m1
Col IV A2	Life Technology	Hs01098873_m1
CRYAB	Life Technology	Hs00157107_m1
FN	Life Technology	Hs00365052_m1
GAPDH	Life Technology	Hs02758991_g1
Lam A1	Life Technology	Hs01074480_m1
MYOC	Life Technology	Hs00165949_m1
TGF-β1	Life Technology	Hs00998133_m1
TGF-β2	Life Technology	Hs00234244_m1
TGF-β3	Life Technology	Hs01086000_m1
TIMP3	Life Technology	Hs00165949_m1

Supplementary Table S3: Antibodies Used for Immunostaining (IF) and Western Blot

**Antibodies**	**Vender**	**Source**	**Catalog number**
AnkG	Invitrogen	Mouse	33-8800
AQP1	ThermoFisher	Rabbit	MA5-32593
αSMA	Dako	Mouse	M0851
β-actin	Sigma	Mouse	A5441

CHI3L1	R&D Systems	goat	AF2599
Fibronectin	R&D Systems	Mouse	MAB19182
FAK	BD Biosciences	Mouse	610087
MGP	Santa Cruz	Mouse	Sc-81546
Myocilin	Proteintech	Mouse	60357-1-Ig
P120Ras GAP	Santa Cruz	Mouse	Sc-63
p-FAK(Y397)	Cell signaling	Rabbit	3283S
p-GRF-1 (Y1105) 190RhoGAP	Invitrogen	Rabbit	PA5-36713
p-RhoA (S188)	Abcam	Rabbit	Ab41435
p-Smad2/3	Cell signaling	Rabbit	8828S
p-Src (Y416)	Cell signaling	Rabbit	2113S
p190-A RhoGAP	Cell signaling	Rabbit	2860S
Rho A	Cell signaling	Rabbit	2117S
Src	Cell signaling	Mouse	2110S

Supplementary Table S4: Kits Used for ELISA

**Kits**	**Vender**	**Catalog number**
Human TGF-β1	R&D Systems	DB100B
Human TGF-β2	R&D Systems	DB250
Human TGF-β3	R&D Systems	DY243

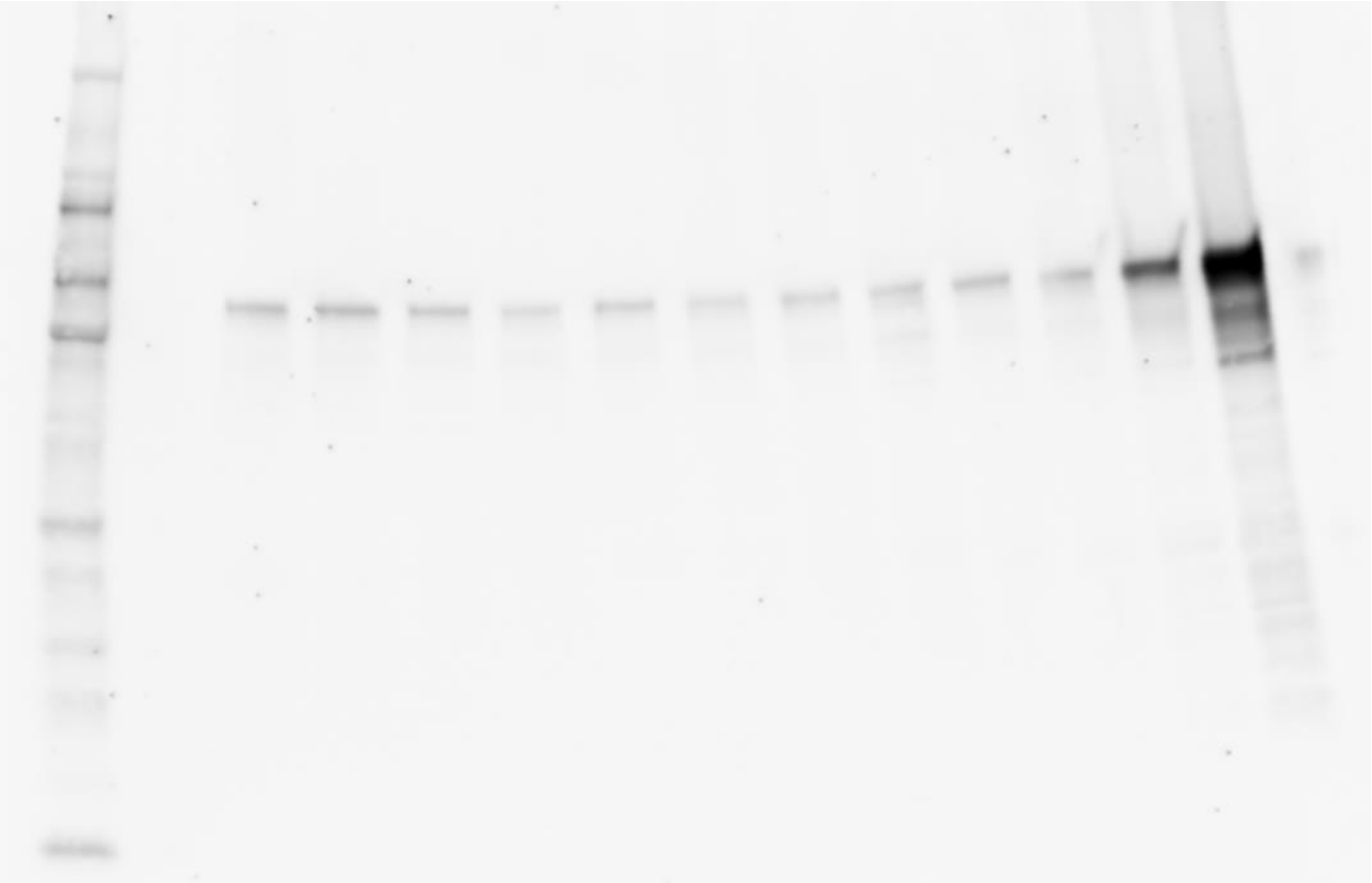
-pFAK(Y397)

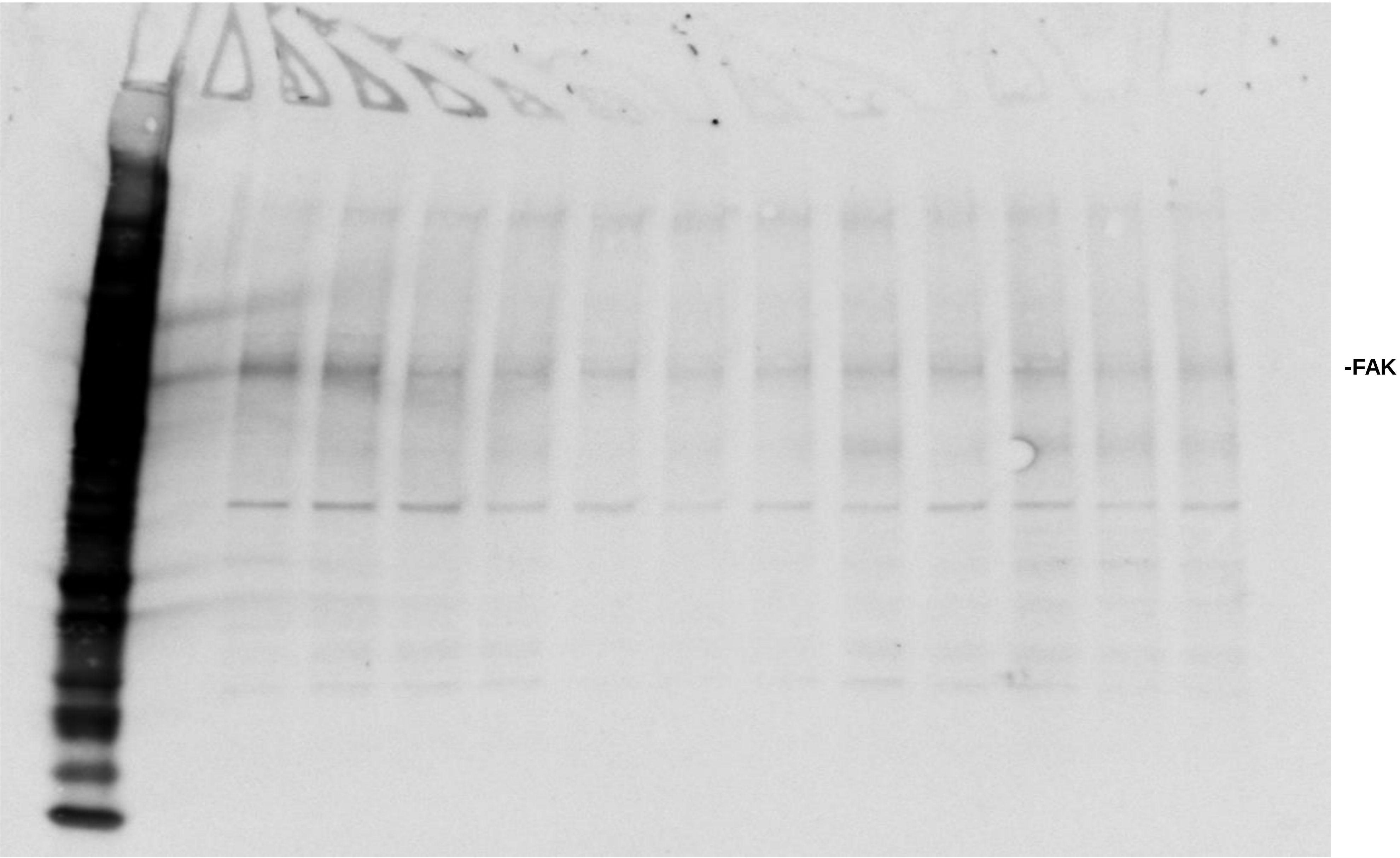
-FAK

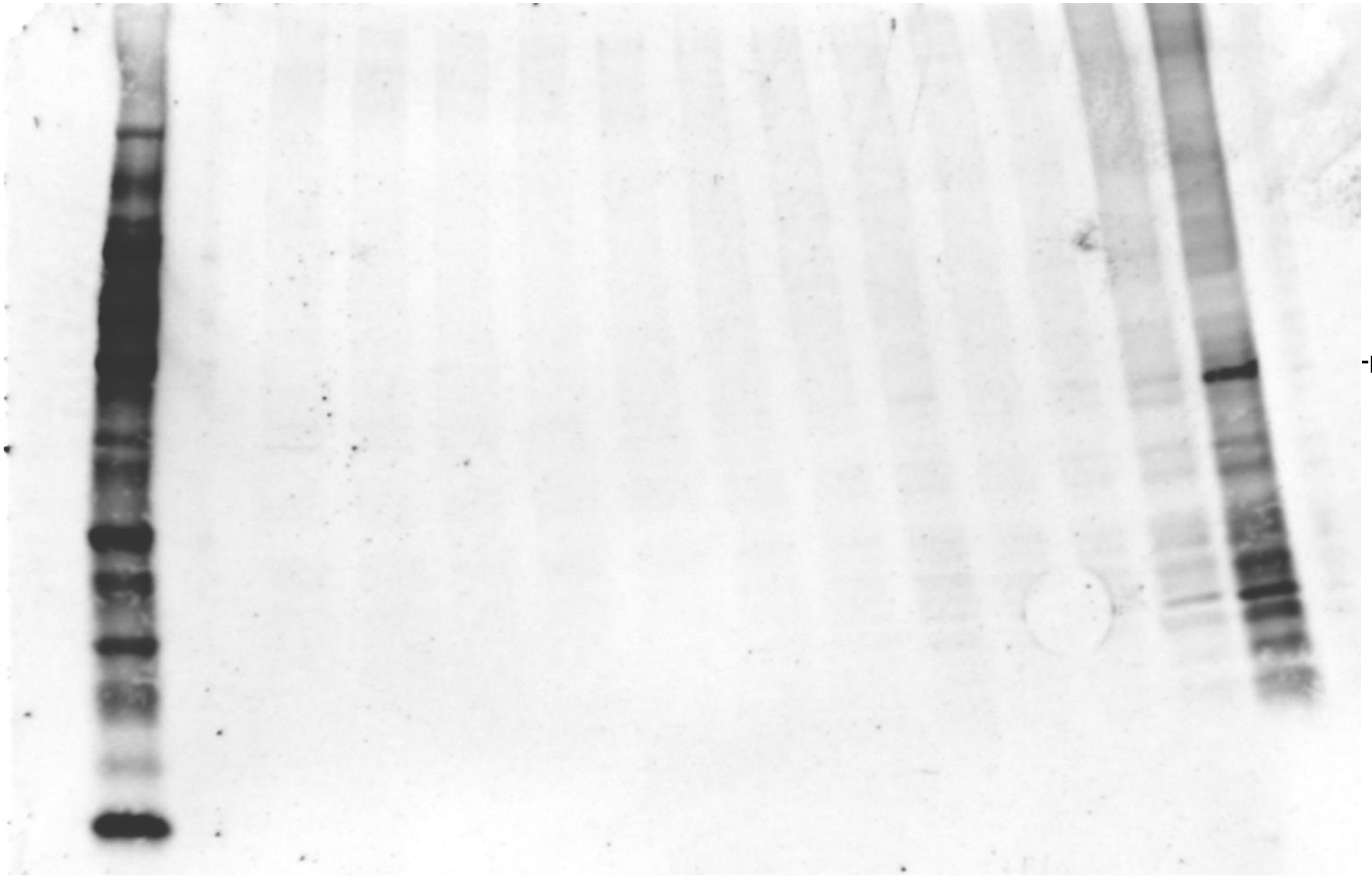

-pSrc (Y416)

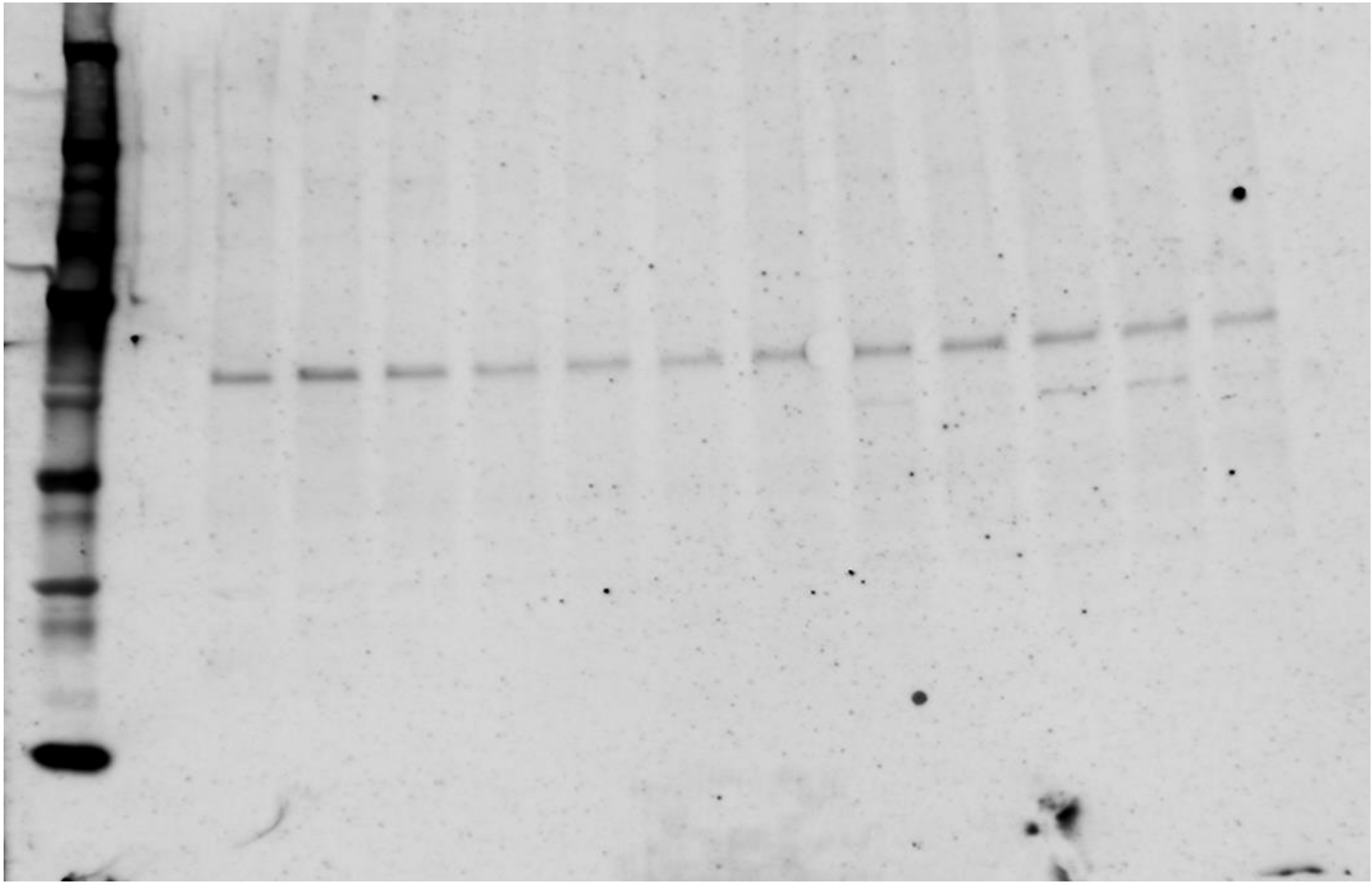
-Src

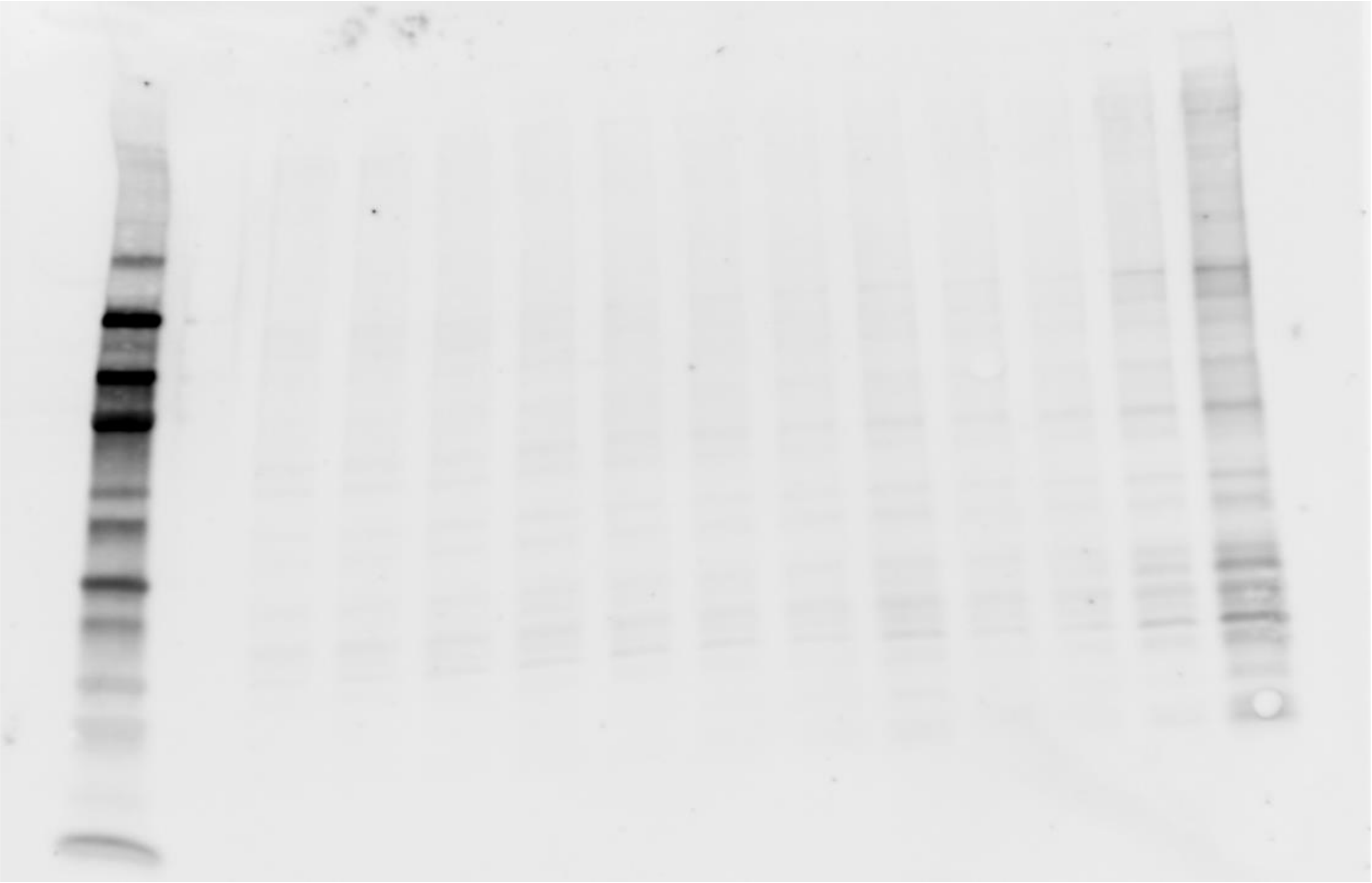
-pP190RhoGAP (Y1105)

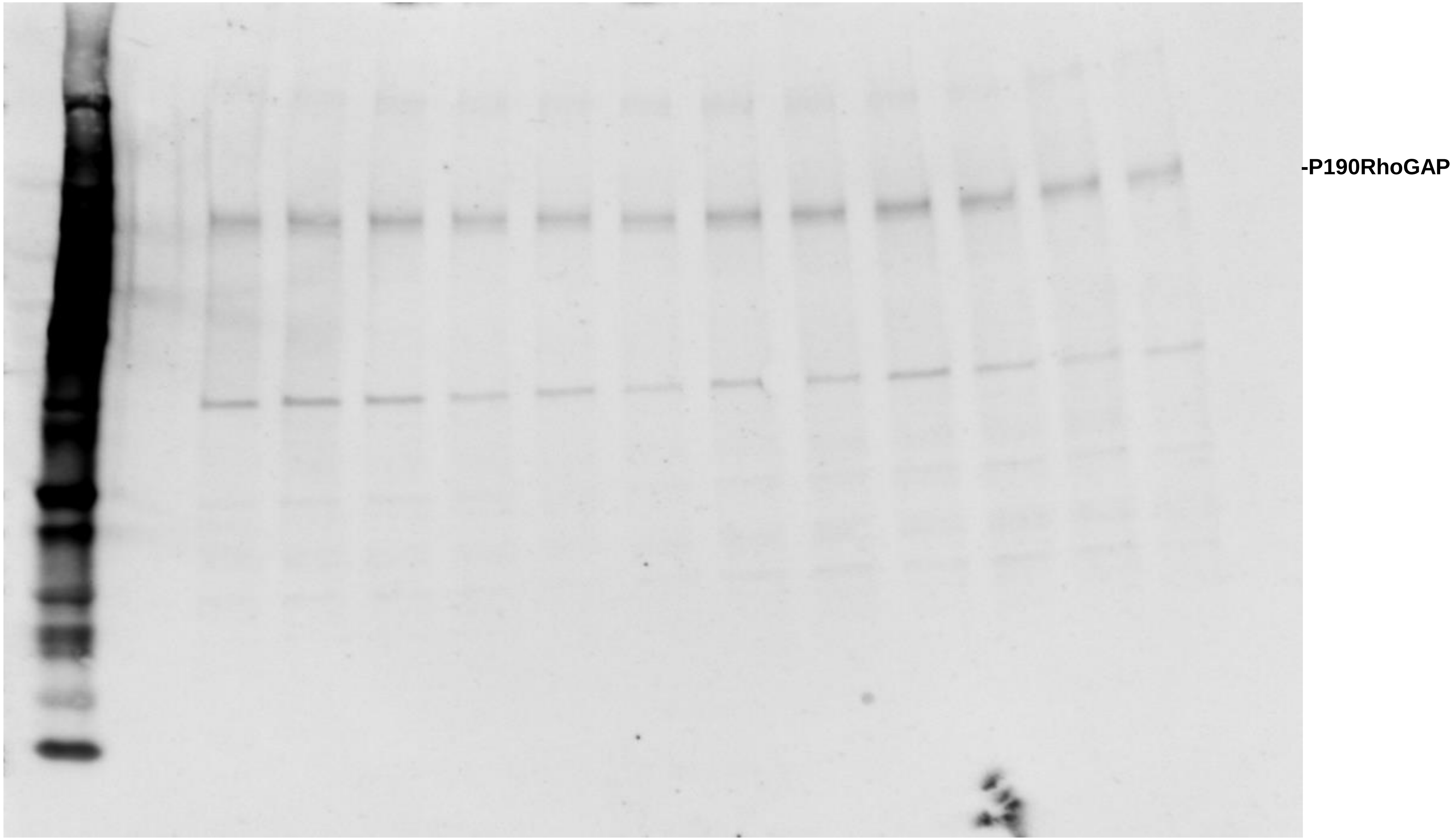
-P190RhoGAP

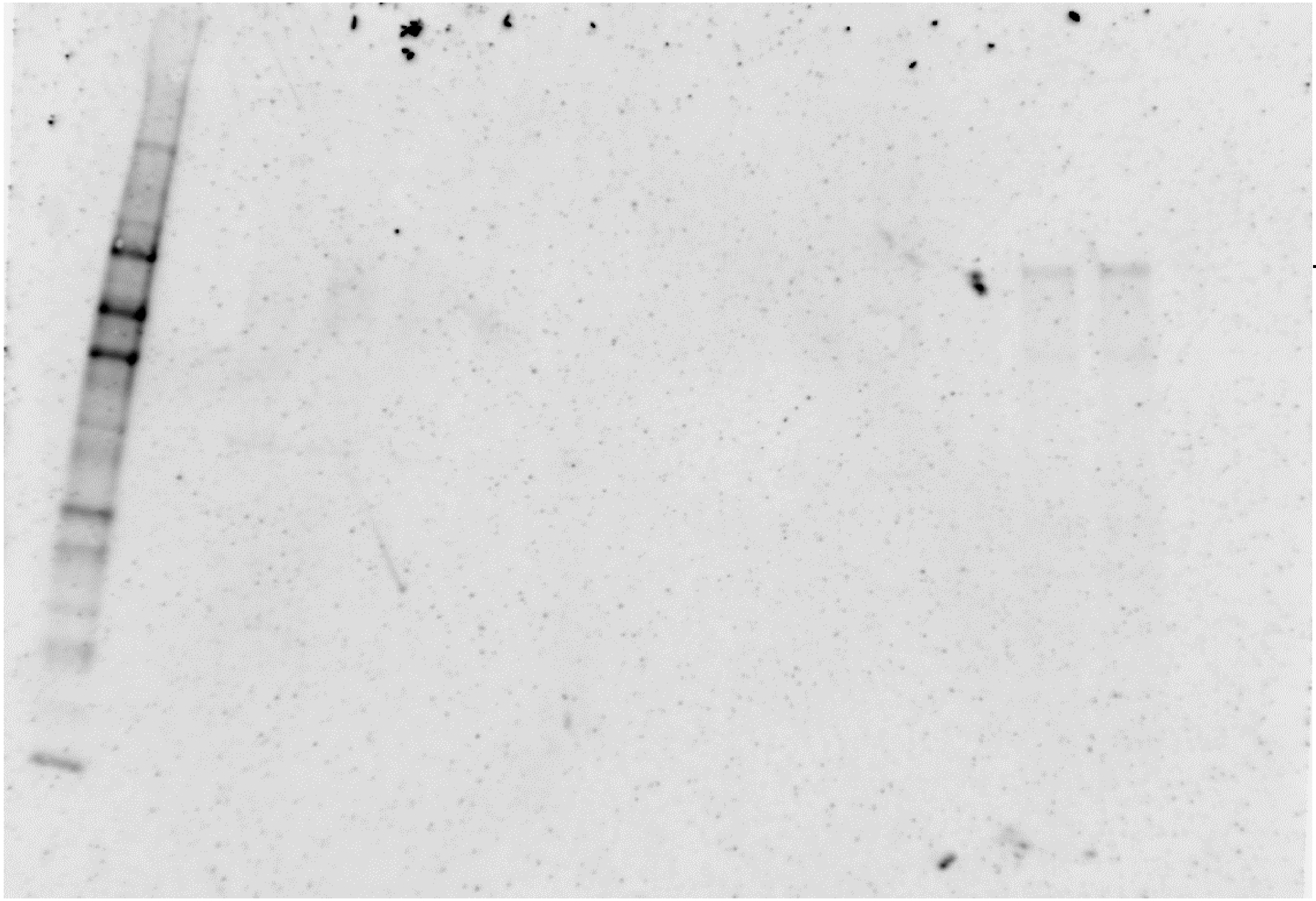

-P120RasGAP

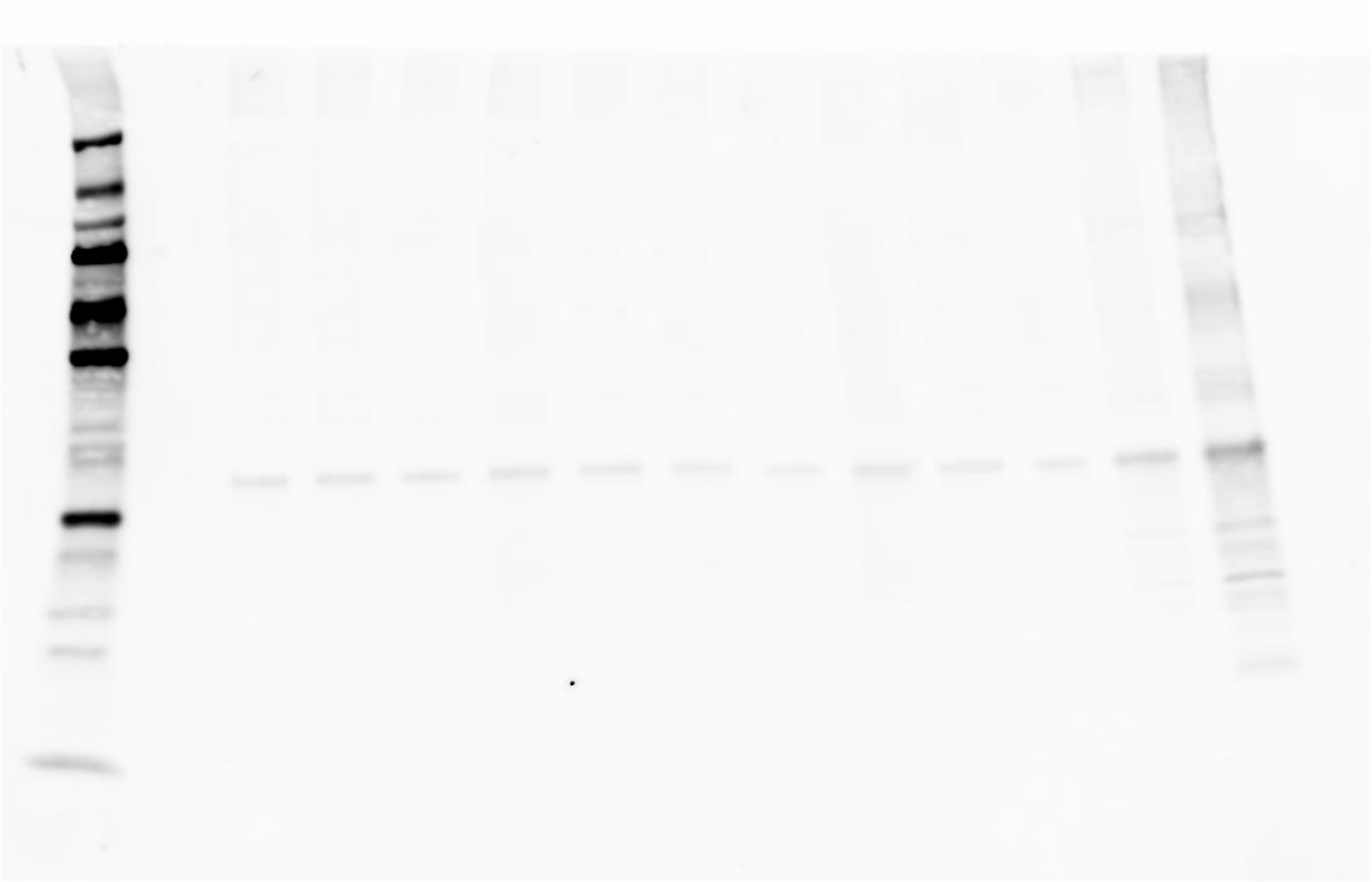

-pRhoA(S188)

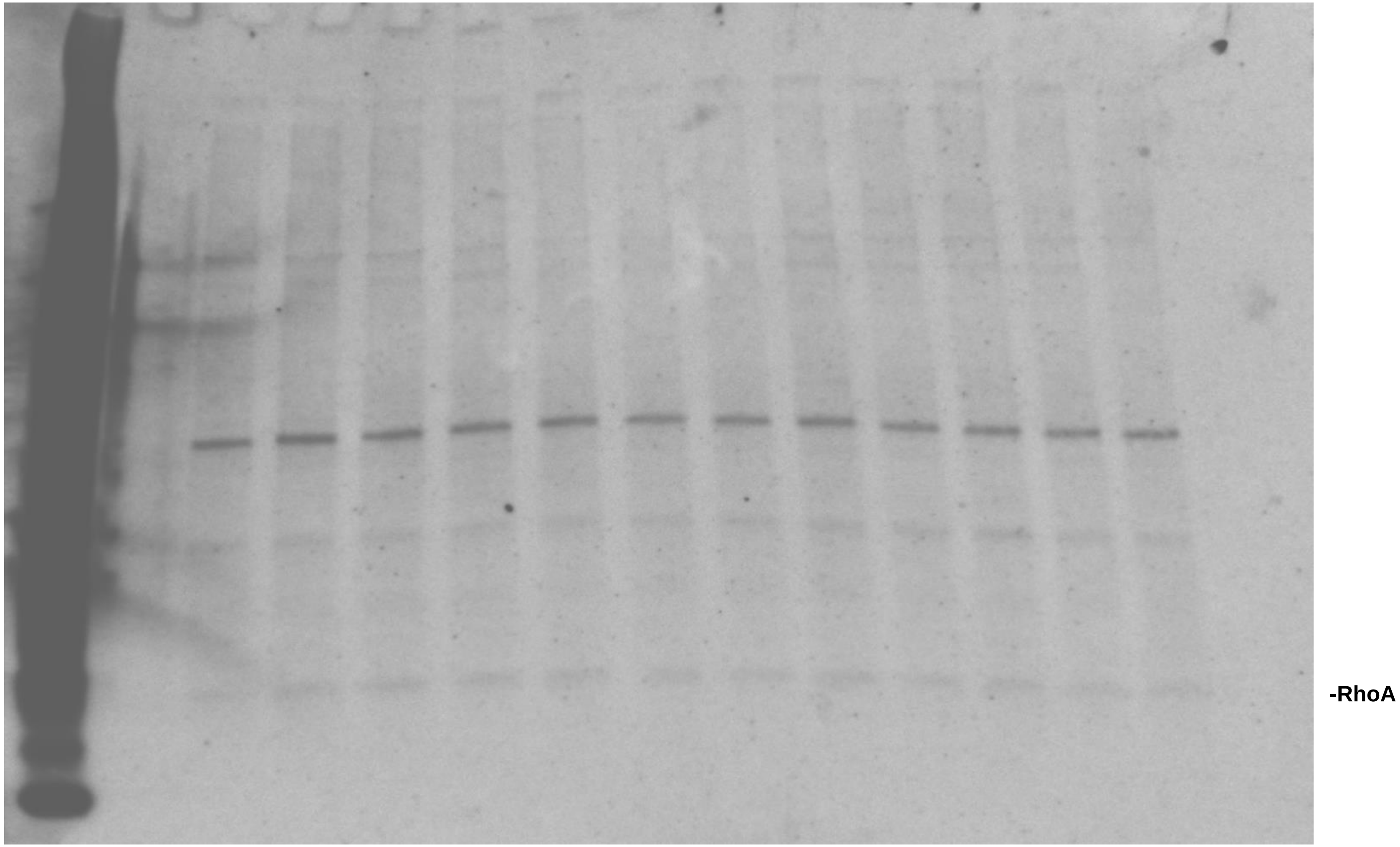
-RhoA

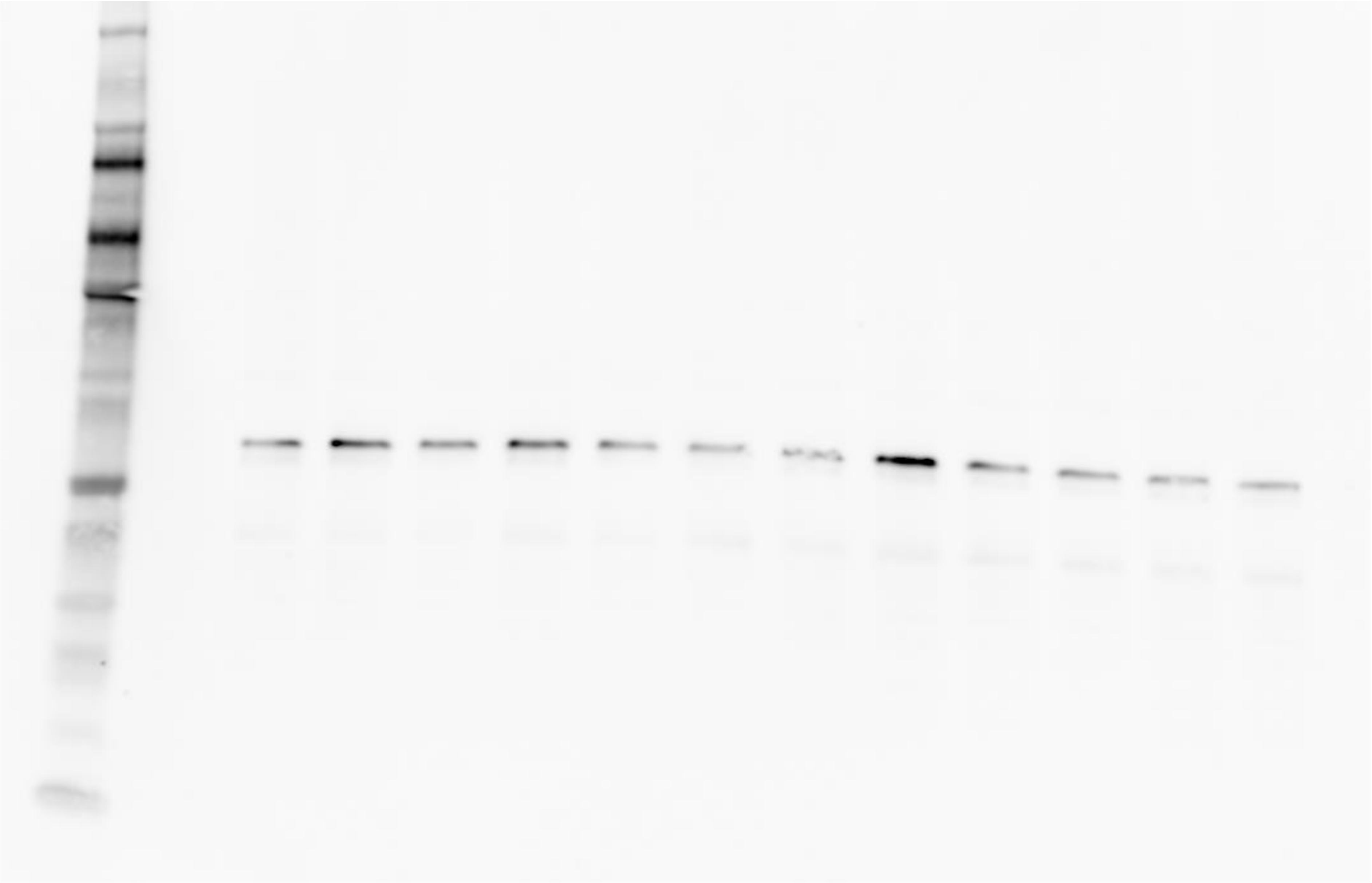
-β-actin

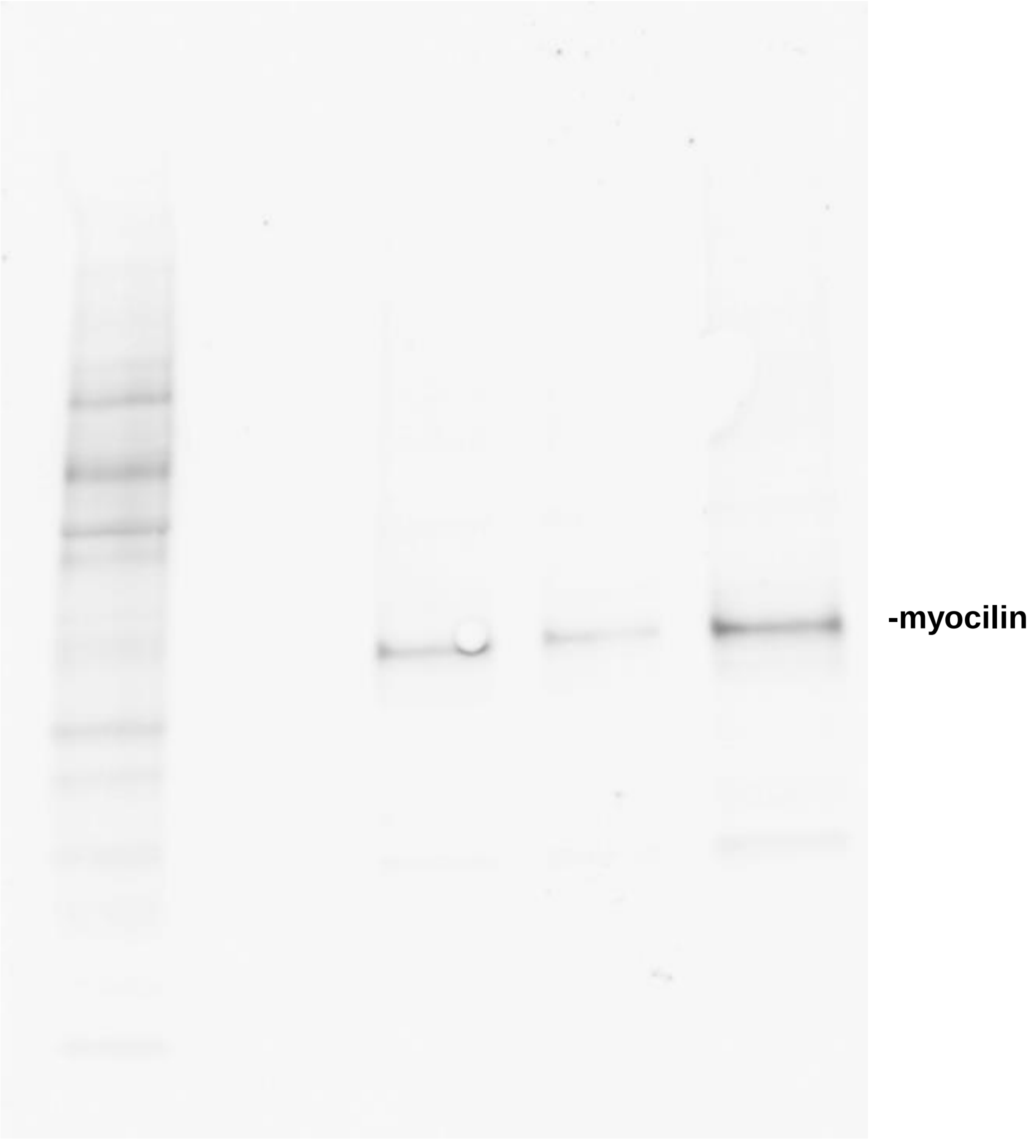
-myocilin

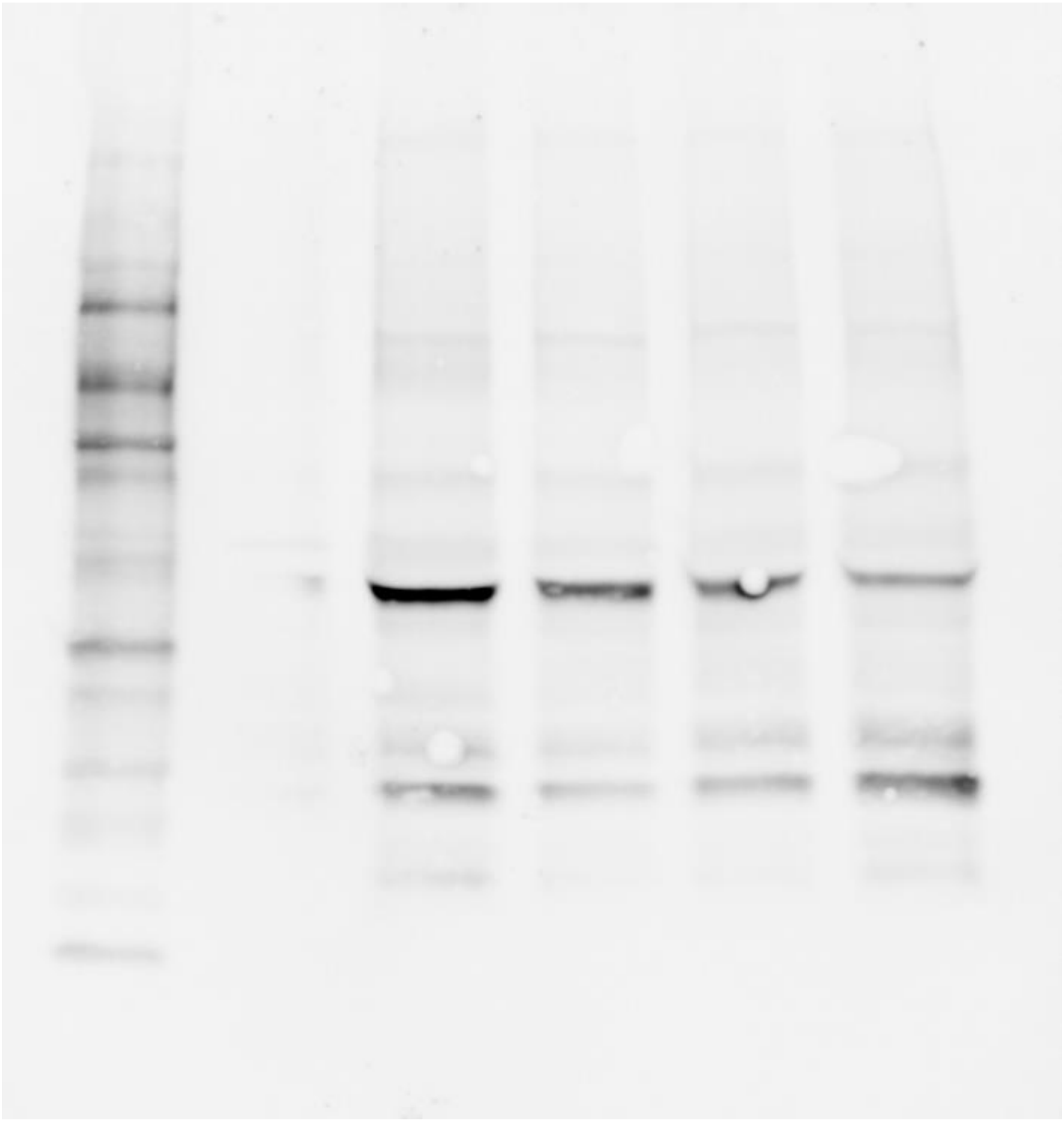
-β-tubulin
